# Hydrophilic CO-Releasing Material of PEGlyated Ruthenium Carbonyl Complex

**DOI:** 10.3390/ma15103597

**Published:** 2022-05-18

**Authors:** Xiao Zhang, Nan Guo, Shuhong Yang, Huma Khan, Weiqiang Zhang

**Affiliations:** 1 Key Laboratory of Applied Surface and Colloid Chemistry Ministry of Education (MOE), School of Chemistry and Chemical Engineering, Shaanxi Normal University, Xi’an 710062, China; zx@snnu.edu.cn (X.Z.); ysh2013snnu@163.com (S.Y.); huma7662@gmail.com (H.K.); 2 School of Chemical Engineering, Northwest University, Xi’an 710127, China; 13991393343@163.com

**Keywords:** ruthenium complex, carbon monoxide releasing molecule, hydrophilicity, PEGylation

## Abstract

The poor water-solubility and instability of Ru(II) carbonyl complex hamper the therapeutic application as CO releasing materials (CO-RMs). To enhance the hydrophilicity and bio-utility of CO, a robust Ru(I) carbonyl sawhorse skeleton was grafted with water-soluble PEGylated sidearm. In this case, 12 PEGylated sawhorse Ru_2_(CO)_4_ complexes were prepared with satisfactory yields and characterized by IR and ^1^H- and ^13^C- NMR. X-ray diffraction analysis of CO-RM **8**, **13** and **14** revealed the featured diruthenium sawhorse skeleton and PEGylated axial ligands. The flask-shaking method measures the water-solubility of CO-RMs, indicating that both bridging carboxylate ligands and PEGlyated axial ligands regulate the hydrophilicity of these CO-RMs. Under photolysis conditions, CO-RM **4**–**13** sustainable released therapeutic amounts of CO in the myoglobin assay. The correlation of the CO release kinetics and hydrophilicity of CO-RMs demonstrated that the more hydrophilic CO-RM released CO faster. The biological test found that the low cytotoxic CO-RM **4** showed a specific anticancer activity toward HT-29 tumour cells.

## 1. Introduction

Recently, biological experiments using transition metal carbonyl complexes as CO-releasing molecules (CO-RM) [1,2] revealed the therapeutic effects of endogenous CO. These effects include anti-inflammatory function, vasodilatation, anti-apoptotic, anti-proliferative, and anti-hypoxia [3]. The organ protection of CO is desirable and attractive because controlling a low concentration of CO indeed protects donor tissues from ischaemia-reperfusion injury [4]. The ruthenium carbonyl complex, [Ru(CO)_3_Cl_2_]_2_, CO-RM-02, mainly were fascinating because it attenuated acute hepatic ischemia-reperfusion injury in rats by reducing serum AST/ALT levels and improved the liver histology score [5,6]. However, the poor solubility of CO-RM-02 and its unregulated CO releasing property in aqueous systems hinder its therapeutic application under physiological conditions [7,8]. To solve the water-solubility issue, CO-RM-02 was solubilized in DMSO, which readily reacts with the CO-RM dimer to generate DMSO-ligated monomeric ruthenium carbonyl species [9]. Motterlini and Mann found that glycinate ligands chelate to the Ru (II) carbonyl moiety, and the corresponding Ru(II) complex (CO-RM-03) was water- soluble. Unfortunately, CO-RM-03 degrades rapidly in human plasma, and the half-life of CO release is only 3.6 min [10]. In fact, due to the intrinsic hydrophobic nature of CO, most metal carbonyl complexes have minimal solubility in an aqueous solution. Although a few ionic transition metal carbonyl complexes are water-soluble, the CO release tests indicated that the M-CO bonds of simple ionic CO-RM degraded quickly under complicated physiological conditions and produced unpredictable side effects, such as blocking blood vessels and causing cytotoxic effects. Increasing the water-solubility and finely controlling the CO release kinetics of transition metal complexes are challenges in therapeutic CO-RMs [11,12,13,14,15].

**Scheme 1:**
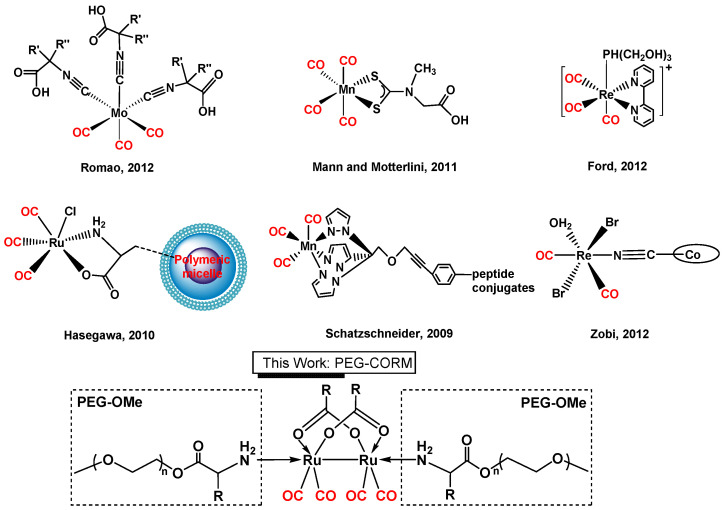
Selected Water-soluble CO-RMs.

Introducing the hydrophilic functional groups into the coordination sphere of transition metal complexes is an efficient way to enhance the water-solubility of the leading CO-RM structure (Scheme 1). The hydrophilic auxiliary ligands bearing carboxylic acid groups improve the hydrophilicity of (Mo(CO)_3_(CNCR′R″CO_2_R‴)_3_ [16] and [Mn(CO)_4_{S_2_CNMe(CH_2_CO_2_H)}] [17], respectively. The classic water-soluble phosphine ligand, P(CH_2_OH)_3_ coordinates to Ru(I) center and significantly enhances the solubility of Photo-CO-RM, 2, 2′-bipyridine tricarbonyl rhenium(I) in PBS solution [18]. Conjugation of transition metal carbonyl moiety with the hydrophilic biomacromolecules also improves the water solubility and biocompatibility of CO-RMs. The micellar bearing [RuCl(glycinate)(CO)_3_] [19], peptide conjugates of [Mn(CO)_3_(tmp)]^+^ [20], and a photo-CO-RM based on vitaminB12, namely, B12-ReCO-RM2 [21] were fabricated to deliver CO in the aqueous system. However, the synthesis and purification of these hydrophilic CO-RMs are sophisticated. More importantly, the CO release kinetics of most of the water-soluble CO-RMs are unpredictable and thus cannot satisfy the basic requirements of ADME proprieties for CO pre-drugs [22]. Polyethylene glycol (PEG) is a non-ionic polymer that improves water solubility and selective drug absorption. Inspired by the idea of the PEG-polymer linking drug system proposed by H. Ringsdorf [23], and the subsequent precedents of PEGlyated therapeutic agents by A. Abuchowski [24]. Herein, CO-RMs, which are PEGlylated Sawhorse Ru carbonyl complexes, were synthesized by incorporating a robust CO-RM leading structure [Ru_2_(CO)_4_(COOR)_2_] with functionalized PEG chains. X-ray single-crystal analysis revealed that the designed PEG esters of amino acids coordinate to Ru(I) of the sawhorse CO-RM lead structure. From the logP measurements and myoglobin assay experiments, it was found that these hydrophilic CO-RMs show a well-controlled CO release property with broad kinetics under physiologic conditions.

## 2. Results and Discussion

**Scheme 2:**
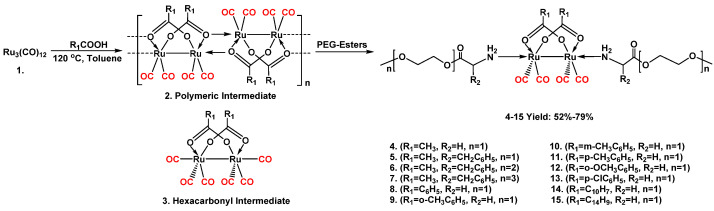
PEGylating of Sawhorse Ru_2_(CO)_4_ complex of **4**–**15**.

Since 1969 when J. Lewis first reported that Ru_2_(CO)_4_(O(C=O)R)_2_L_2_, features a sawhorse structure [25], a considerable number of the ruthenium(I) carbonyl complexes have been prepared and characterized. However, the inherited poor water-solubility and biological incompatibility of sawhorse ruthenium complexes are obstacles to their application in in the biological system [26]. To increase the hydrophilicity of these ruthenium carbonyl complexes, the water-soluble amino acid glycol and PEG esters were tethered at the axial position of the sawhorse structure, respectively (Scheme 2). The thermolysis of Ru_3_(CO)_12_ **1** in acetic acid affords polymeric sawhorse ruthenium carbonyl **2**, and the sequential ligand substitution reaction using glycine methyl esters flourishes the PEGylated sawhorse complexes **4**–**7** with 61–74% yield. The light-yellow products **4**–**7** are moisture and air-stable easily handled without proof from oxygen or light. The thermolysis of Ru_3_(CO)_12_ **1** and aromatic carboxylic acid at 120 °C in toluene generates hexacarbonyl Ru(I) intermediates **2** and with the characteristic carbonyl bands at 2103 w, 2079 vs, 2035 vs, 2004 vs and 1938 w cm^−1^. The axial carbonyl ligands of **2** are labile and can readily be substituted by glycine esters of ethylene glycol monomethyl ester. Thermolysis using various bridged aromatic carboxylic acids afforded **8**–**15**, and FT-IR, ^1^H-NMR, mass spectrometry and elemental analysis were used to fully characterize complexes **4**–**15**. The IR spectra of the Ru(I) complexes identified the four characteristic carbonyl bands at 2028–1938 cm^−1^ of sawhorse Ru_2_(CO)_4_ complexes whilst the bridged-carboxylate ligand showed C=O band at 1743 cm^−1^. The NMR spectra revealed more detailed information about the molecular structures of these ruthenium complexes with more details. In the ^1^H-NMR spectrum of **4**, the bridged-acetate was observed as a singlet at δ 1.94 ppm, and the protons of NH_2_ appeared as a singlet at δ 2.90 ppm. CH_2_ and OCH_3_ of axial ethylene glycol monomethyl ester glycine ester appeared as triplets at δ 4.35, 3.75, 3.62 ppm, and a singlet at 3.39 ppm, respectively. The ^13^C{^1^H} NMR spectrum of **4** shows three types of CO resonances, and the Ru bonded carbonyl groups are at δ 204 ppm, the bridging carboxylate is at δ 172 ppm, and the ester group is at δ 184 ppm, respectively. **4**–**15** showed similar resonances for both bridged and axial ligands. Notably, the amino protons of μ_2_-acetato complex **4** appear at δ 2.90 ppm, lower than the corresponding chemical shift of μ_2_-arylcarboxylato complexes **8**–**15**. The shielded amino proton of **4** reflects less electron donation to the sawhorse unite, indicating the corresponding axial ligand may be more labile.

**Figure 1:**
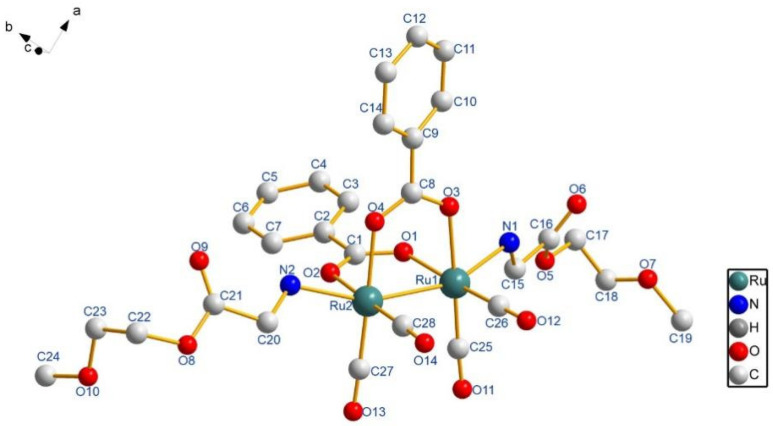
Molecular structures of **8** (H atoms are omitted for clarity).

Yellow crystals of three diruthenium (I) complexes **8** (Figure 1), **13** and **14** were obtained via the diffusion of petroleum ether to CH_2_Cl_2_ solution of the complexes at 0 °C. Single crystal X-ray diffraction was used to analysis confirmed the molecular structures of these complexes, and the selected bond distances and angles are shown in Table 1. The molecular structures feature a typical sawhorse structure that consists of a diruthenium tetracarbonyl core surrounded by two ethylene glycol monomethyl ester glycine esters as axial ligands and two arylcarboxylate ligands at equatorial positions. Three crystals belong to the monoclinic system with the C2/c space group. The Ru-Ru bond distances in these sawhorse skeletons are 2.6694(10) Å (**8**), 2.6634(7) Å (**13**) and 2.6727(5) Å (**14**), respectively; these values are with a metal-metal single bond [27]. The Ru-CO bond length of each terminal carbonyl is slightly different. For instance, the average Ru-CO bond length of these complexes is about 1.83 Å, which is shorter than Ru-CO (1.943(3) Å and 1.903(3) Å) of CO-RM-3 [10], but longer than Ru-CO(1.76 Å) in those of axial triphenylphosphine analogues [27]. In complex **8**, Ru(1)-N(1) 2.239(5) Å is slightly more than Ru(2)-N(2) 2.210(5) Å, and the average Ru-Ru-N angle is 158°, indicating the former axial ligand might be more labile and readily dissociate during the CO releasing process. Interesting, in complex **13**, Ru(1)-N(1) and Ru(2)-N(2) with same distance at 2.249(4) Å, but Ru(2)-Ru(1)-N(1) = 160.28(11)° bigger than Ru(1)-Ru(2)-N(2) = 159.09(11)°.

**Figure 2:**
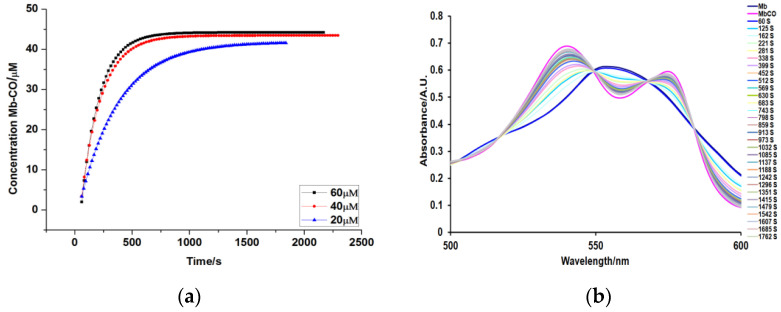
Photo-activated CO release profile for **4** (**a**) UV-vis spectrum showing the Q-bands during the conversion of deoxy-Mb to Mb-CO with time while the concentration of CO-RMs is 60; (**b**) The CO-releasing kinetics of **4** in which [Mb-CO] was plotted with CO-RM at 60, 40, 20 µM against time.

The CO release activity of each CO-RM in vivo was measured with the “golden standard” of myoglobin assay. Firstly, sodium dithionite was added to reduce myoglobin to deoxy-myoglobin (deoxyMb) in PBS (pH = 7.4) at 37.8 °C. A stock solution of CO-RM was added and then activated by LED-UV radiation at 365 nm, releasing CO in vivo. The change of deoxyMb to carbonmonoxy-myoglobin (MbCO) was monitored by UV-vis spectroscopy. A typical series of electronic absorption spectra (Figure 2) showed the conversion of deoxyMb to MbCO in the presence of CO released from **4**. The half-lives of CO release rate of **4** at 60 µM is 166 s, 40 µM is 172 s, and 20 µM is 267 s. Four isosbestic points demonstrated the biocompatibility of this CO-RM. The dark-controlled CO release experiment showed that all CO-RMs are stable and do not spontaneously degrade under physiological conditions. Tuning the time and density of UV radiation also control the CO releasing kinetics from CO-RM whilst the molecular structures of CO-RMs determine their photo-sensitivity and CO release activity.

To identify the structural features of CO-RMs that govern the CO releasing behaviour, the CO release kinetics of **4**–**15** were correlated to the corresponding M-CO band and lipophilicity in Table 2. Firstly, the oil-water partition coefficient logP values of complexes were measured by the “flask-shaking” method with n-octanol and water, respectively [28]. The logP value of **4**–**13** ranges from 0.39 to 1.78. The water-solubility of these sawhorse ruthenium complexes mainly depend on both the axial glycol amino esters and bridging carboxylate, respectively. As shown in Table 2, the more hydrophilic CO-RMs release carbon monoxide faster. **4** exhibited the lowest logP value of 0.39, which release CO fastest and convert 30 μm Mb to MbCO for just 163 s using 60 μm CO-RM. Since the benzyl substitutes of axial ligand significantly reduce the hydrophilicity of **5**, The logP value of **5** increased to 1.41 and the CO release half-life of **5** t_1/2_, 60 μm decreased to 276 s. With the increase in the degree of polymerization of PEG chain, it is conducive to improving the water solubility of CO-RM **5**–**7**, and the CO release rate increases with t_1/2_, 60 μm from 276 s to 189 s.

The substitutes on the arene are related to the hydrophilicity of aromatic carboxylate bridging CO-RM. **8**, **9** and **12** showed higher logP values of 1.71, 1.67 and 1.78, respectively, which released CO much slower with t_1/2_,60 μm around 1000 s. Interestingly, The para-substituted methyl-, chloro- **10**, **11** and meta methoxy- **13** groups increase the hydrophilicity and the corresponding CO rate of each aromatic CO-RM. **14** and **15** are too hydrophobic to be evaluated via the “flask-shaking” method, in which t_1/2_, 60 μm is over 2000s. Compared with compounds **4**, **8**–**15** with benzyl H atom and polymerization degree *n* = 1, the structure of acetic acid bridging is better than that of aryl acid bridging. The methyl and methoxy substituents on the benzene ring can improve the water solubility to a certain extent, but the increase in the chain length of bridged alkyl carboxylic acid will significantly reduce the water solubility. Therefore, acetic acid as bridging ligand and hydrophilic PEGlyated ester are essential to improving the water solubility of CO-RM and accelerating the release of CO.

**Figure 3:**
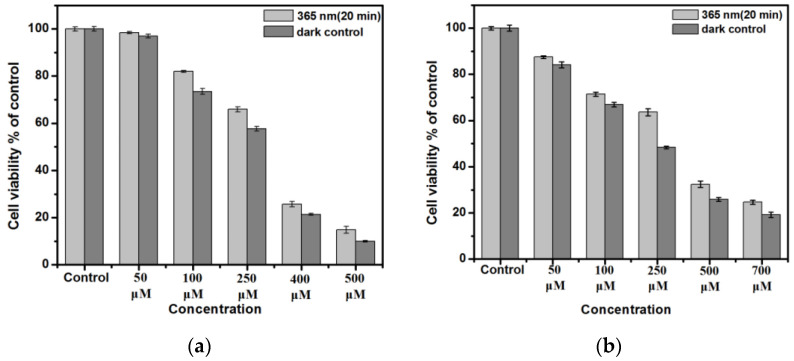
Cell viability of RAW264.7 cell (**a**) and HT29 (**b**) in presence of **4**. Cells were grown in the presence of **4** (50–500 μM) and right in the dark or irradiated at 365 nm for 20 min.

Each component of CO-RM, such as Ru, carboxylic acid and amino acid esters, is considered non-toxic than most of the other CO-RMs. The cytotoxicity of CO-RM is still unknown. To evaluate the bioactivity of PEG-CORM with different functional groups, we investigated the cytotoxicity of **4** and **8** with the murine macrophage cell line, RAW 264.7 and the human colon adenocarcinoma cell line, HT29. Generally, IC50 of **4** over two cell lines showed less cytotoxicity in the dark, in constant with the cellular protection effect of endogenous CO. The MTT experiments showed that 100 µM to 500 µM of **4** significantly impacted the cell viability of RAW264.7 (Figure 3a), IC50 value is 253.3 µM. 100 µM of **4** started to reduce the cell survival rate. As the concentration of CO-RM increased, the survival rate of the cell dropped sharply. In the presence of 400 µM and 500 µM of **4**, RAW264.7’s survival rate was 22.3% and 10%, respectively. Due to its limited water-solubility, the MTT experiments of **8** were carried out at 10–50 µM (Figures S17 and S18). **8** showed little effect on RAW264.7 cell viability at a concentration of 10 µM, 99.6%. The survival rate of RAW 264.7 cells decreased slightly with the increase in concentration; 50 µM of **8** decreased to 94.6%, similar to the toxicity of **4**.

The human colon adenocarcinoma cell line HT29 received particular interest in studies focused on food digestion and bioavailability due to its ability to express characteristics of mature intestinal cells. To evaluate the potential of PEG-CO-RMs for CO therapy as anticancer agents, we measured the cytotoxicity of **4** and **8** over HT-29. Experiments with HT29 cells used the concentration of **4** in the range from 50 µM to 700 µM (Figure 3b), and **8** in the range of 10–50 µM (Figure S19). At a concentration of 50 µM **4** and **8**, 12.5% and 6.65% of the cells lose activity in light stimulation, respectively. Compound **4** showed a better inhibitory effect on cancer cells, indicating that alkyl bridged carboxylic acid ligands with higher toxicity than aryl carboxylic acid ligands. Moreover, when the concentration increased to 700 µM, HT29 cells survived just 24.7%. Interestingly, **4** showed similar anticancer activity in the dark, indicating the anticancer activity of **4** might result from PEG-CO-RM as a whole rather than its’ released CO.

## 3. Materials and Methods

All manipulations were accomplished with standard Schlenk techniques. Decacarbonyl-ruthenium (Ru_3_(CO)_12_) and mPEG amino acid esters were prepared according to literature procedures [27]. CO releasing test were performed using myogblin assay. The cytotoxicity and anticancer activity were measured with RAW264.7 and HTC-29 cells, respectively. The details of experiments were listed in ESI.

## 4. Conclusions

In conclusion, the robust sawhorse skeletons of the diruthenium carbonyl complex were devised with PEGylated ligands to tune the CO releasing and bioavailability of CO-RMs. The myoglobin assay test on the CO releasing rate showed well-controlled release kinetics of CO-RMs **4**–**13** with t_1/2_, 60 μM from 166 s to 2699 s. The logP values of CO-RMs were correlated with CO release rates, revealing the intrinsic relationship between CO-RMs’ water-solubility and CO releasing activity. The CO-RMs with smaller logP released CO faster, which might prove the concept of enhancing water-solubility to improve the CO release properties. The hydrophilicity of CO-RM was finely tuned via selecting carboxylate bridging ligands and glycol amino acid esters. MTT assay confirmed that CO-RM 4 consisted of acetate and glycol glycine ester less cytotoxic to RAW264.7, but toxic to HT29 cancer cells. These CO releasing and bioactivity experiments demonstrated the PEGylated Sawhorse ruthenium carbonyl complex’s drug-like properties and the promising therapeutic potential.

## Figures and Tables

**Table 1:** Selected Bond Distances (Å) and Angles (deg) for **8**, **13** and **14**.

Entry	Ru(1)–Ru(2)	Ru(1)–N(1)	Ru(2)–N(2)	Ru(1)–Ru(2)–N(2)	Ru(2)–Ru(1)–N(1)
8	2.6694(10)	2.239(5)	2.210(5)	157.86(15)	158.27(15)
13	2.6634(7)	2.249(4)	2.249(4)	159.09(11)	160.28(11)
14	2.6727(5)	2.233(4)	2.240(4)	158.27(11)	159.84(10)

**Table 2:** The Correlation of Hydrophilicity and CO releasing kinetics of CO-RM.

CO-RM	logP ^a^.	t_1/2_, 60 µM ^b^.
4	0.39	166
5	1.41	276
6	1.17	249
7	1.05	189
8	1.71	1209
9	1.67	632
10	1.03	962
11	1.06	1096
12	1.78	1450
13	1.26	966
14	N. D.	2699
15	N. D.	2472

Note: [a] Oil-water partition coefficient by UV-vis. [b] CO releasing Kinetics measured with myoglobin assay as t_1/2_, s.

## Data Availability

Data are contained within the article.

## Supplementary Materials

The following are available online at https://www.mdpi.com/article/10.3390/ma15103597/s1, Figures S1 and S2: Molecular structure of complex **13**, **14**. Tables S1 and S2: Data collection and structural refinements details for single-crystal X-ray diffraction studies of complexes **8**, **13** and **14**, and selected bond lengths (Å). Figure S3: NMR spectra of complex **4**–**15**. Figure S4: Photo-activated CO release profile for **4**–**15.** Table S3: Stand curve measurement and calculation of logP. Figure S5: UV-vis spectrum showing the Q-bands during different concentrations of **4** (left) and Standard curve of **4** (right). Figure S6: UV-vis spectrum showing the Q-bands during different concentrations of **5** (left) and Standard curve of **5** (right). Figure S7: UV-vis spectrum showing the Q-bands during different concentrations of **6** (left) and Standard curve of **6** (right). Figure S8: UV-vis spectrum showing the Q-bands during different concentrations of **7** (left) and Standard curve of **7** (right). Figure S9: UV-vis spectrum showing the Q-bands during different concentrations of **8** (left) and Standard curve of **8** (right). Figure S10: UV-vis spectrum showing the Q-bands during different concentrations of **9** (left) and Standard curve of **9** (right). Figure S11: UV-vis spectrum showing the Q-bands during different concentrations of **10** (left) and Standard curve of **10** (right). Figure S12: UV-vis spectrum showing the Q-bands during different concentrations of **11** (left) and Standard curve of **11** (right). Figure S13: UV-vis spectrum showing the Q-bands during different concentrations of **12** (left) and Standard curve of **12** (right). Figure S14: UV-vis spectrum showing the Q-bands during different concentrations of **13** (left) and Standard curve of **13** (right). Figure S15: RAW264.7 cell changes inhibited by **4** (dark: a-50 μM; b-500 μM, illumination: c-50μM; d-500 μM). Figure S16: HT29 cell changes inhibited by **4** (dark: a-50 μM; b-500 μM, illumination: c-50μM; d-500 μM). Figure S17: RAW264.7 cell changes inhibited by **8**. Figure S18: HT29 cell changes inhibited by **8**. Figure S19: Cell viability of RAW264.7 cell and HT29 in presence of **8**. References [29,30,31] are cited in the supplementary materials.

## References

[1] Motterlini R., Otterbein L.E. (2010). Selected Reviews of therapeutic application of CO and CO-RM. The therapeutic potential of carbon monoxide. Nat. Rev. Drug Discov..

[2] Bernardes G.J.L. (2014). Carbon-Monoxide-Releasing Molecules for the Delivery of Therapeutic CO In Vivo. Angew. Chem. Int. Ed..

[3] Ryter S.W., Alam J., Choi A.M.K. (2006). Heme Oxygenase-1/Carbon Monoxide: From Basic Science to Therapeutic Applications. Physiol. Rev..

[4] Kim H.J., Joe Y., Yu J.K., Chen Y., Jeong S.O., Mani N., Cho G.J., Pae H., Ryterd S.W., Chung H.T. (2015). Carbon monoxide protects against hepatic ischemia/reperfusion injury by modulating the miR-34a/SIRT1 pathway. Biochim. Biophys. Acta.

[5] Wei Y., Chen P., Bruyn M., Zhang W., Bremer E., Helfrich W. (2010). Carbon monoxide-Releasing Molecule-2 (CO-RM-2) attenuates acute hepatic ischemia reperfusion injury in rats. BMC Gastroenterol..

[6] Caumartin Y., Stephen J., Deng J., Lian D., Lan Z., Liu W., Garcia B., Jevnikar A.M., Wang H., Cepinskas G. (2011). Carbon monoxide-releasing molecules protect against ischemia-reperfusion injury during kidney transplantation. Kidney Int..

[7] Kretschmer R., Gessner G., Görls H., Heinemann S.H., Westerhausen M. (2011). Dicarbonyl-bis(cysteamine)iron(II): A light induced carbon monoxide releasing molecule based on iron(CO-RM-S1). J. Inorg. Biochem..

[8] Romanski S., Kraus B., Schatzschneider U., Neudörfl J.M., Amslinger S., Schmalz H.G. (2011). Acyloxybutadiene Iron Tricarbonyl Complexes as Enzyme-Triggered CO-Releasing Molecules (ET-CO-RMs). Angew. Chem. Int. Ed..

[9] Lomont J.P., Nguyen S.C., Harris C.B. (2014). Exploring the Utility of Tandem Thermal–Photochemical CO Delivery with CORM-2. Organometallic.

[10] Johnson T.R., Mann B.E., Teasdale I.P., Adams H., Foresti R., Green C.J., Motterlini R. (2007). Metal carbonyls as pharmaceuticals? [Ru(CO)_3_Cl(glycinate)], a CO-Releasing molecule with an extensive aqueous solution chemistry. Dalton Trans..

[11] Seixas J.D., Mukhopadhyay A., Santos-Silva T., Otterbein L.E., Gallo D.J., Rodrigues S.S., Guerreiro B.H., Gonçalves A.M.L., Penacho N., Marques A.R. (2013). Characterization of a versatile organometallic pro-drug (CO-RM) for experimental CO based therapeutics. Dalton Trans..

[12] Poh H.T., Sim B.T., Chwee T.S., Leong W.K., Fan W.Y. (2014). The Dithiolate-Bridged Diiron Hexacarbonyl Complex Na_2_[(μ-SCH_2_CH_2_COO)Fe(CO)_3_]_2_ as a Water-Soluble PhotoCO-RM. Organometallics.

[13] Kianfar E., Monkowius U., Portenkirchner E., Knöer G., Naturforsch Z. (2014). Synthesis and Characterization of Novel Re(BIAN)(CO)(3)Cl Derivatives Including the First Example of a Water-soluble Tricarbonyl Rhenium(I) Complex with Bis(imino)acenaphthene Ligands. Z. Naturforsch. B.

[14] Mede R., Klein M., Claus R.A., Krieck S., Quickert S., Görls H., Neugebauer U., Schmitt M., Gessner G., Heinemann S.H. (2016). CO-RM-EDE1: A Highly Water-Soluble and Nontoxic Manganese-Based photoCO-RM with a Biogenic Ligand Sphere. Inorg. Chem..

[15] Zhang W., Atkin A.J., Fairlamb I.J.S., Whitwood A.C., Lynam J.M. (2011). Synthesis and Reactivity of Molybdenum Complexes Containing Functionalized Alkynyl Ligands: A Photochemically Activated CO-Releasing Molecule (PhotoCO-RM). Organometallics.

[16] Marques A.R., Kromer L., Bento I., Otterbein L.E., Blattler W.A., Romao C.C. (2012). Generation of Carbon Monoxide Releasing Molecules(CO-RMs) as Drug Can didates for the Treatment of Acute Liver Injury: Targeting of CO-RMs to the Liver. Organometallics.

[17] Crook S.H., Mann B.E., Meijer A.J.H.M., Adams H., Sawle P., Scapens D., Motterlini R. (2011). [Mn(CO)_4_{S_2_CNMe(CH_2_CO_2_H)}], a new water-soluble CO-releasing molecule. Dalton Trans..

[18] Pierri A.E., Pallaoro A., Wu G., Ford P.C. (2012). A Luminescent and Biocompatible PhotoCO-RM. J. Am. Chem. Soc..

[19] Hasegawa U., Vlies A.J., Simeoni E., Wandrey C., Hubbell J.A. (2010). Carbon Monoxide-Releasing Micelles for Immunotherapy. J. Am. Chem. Soc..

[20] Pfeiffer H., Rojas A., Niesel J., Schatzschneider U. (2009). Sonogashira and “Click” reactions for the N-terminalandside-chain functionalization of peptides with [Mn(CO)_3_(tpm)]^+^-based CO releasing molecules (tpm = tris(pyrazolyl)methane). Dalton Trans..

[21] Zobi F., Blacque O., Robert A., Marcus J., Schaubc C., Bogdanova A.Y. (2012). 17e^−^ rhenium dicarbonyl CO-releasing molecules on a cobalamin scaffold for biological application. Dalton Trans..

[22] Wang P., Liu H., Zhao Q., Chen Y., Liu B., Zhang B., Zheng Q. (2014). Syntheses and evaluation of drug-like properties of CO-releasing molecules containing ruthenium and group 6 metal. Eur. J. Med. Chem..

[23] Ringsdorf H. (1975). Structure and properties of pharmacologically active polymers. J. Poly. Sci..

[24] Pasut G., Sergi M., Veronese F.M. (2008). Anti-cancer PEG-enzymes: 30 Years Old, but Still a Current Approach. Adv. Drug Deliv. Rev..

[25] Crooks G.R., Johnson B.F.G., Lewis J., Williams I.G., Gamlen G. (1969). Chemistry of polynuclear compounds. Part XVII. Some carboxylate complexes of ruthenium and osmium carbonyls. J. Chem. Soc. A.

[26] Therrien B., Suss-Fink G. (2009). Sawhorse-type Diruthenium Tetracarbonyl Complexes. Coord. Chem. Rev..

[27] Yang S., Chen M., Zhou L., Zhang G., Gao Z., Zhang W. (2016). Photo-activated CO-releasing Molecules (PhotoCO-RMs) of Robust Sawhorse Scaffolds [µ_2_-OOCR^1^,η^1^-NH_2_CHR^2^(C=O]OCH_3_,Ru(I)_2_CO_4_]. Dalton Trans..

[28] Glomme A., März J., Dressman J.B. (2005). Comparison of a Miniaturized Shake-Flask Solubility Method with Automated Potentiometric Acid/Base Titrations and Calculated Solubilities. J. Pharm. Sci..

[29] SAINT (2009). Data Reduction Software.

[30] Sheldrick G.M. (2010). SADABS, Program for Empirical Absorption Correction of Area Detector Data.

[31] Dolomanov O.V., Bourhis L.J., Gildea R.J., Howard J.A.K., Puschmann H. (2009). A Complete Structure Solution, Refinement and Analysis Program. J. Appl. Cryst..

