# Supplementary material for: Hydrophilic CO-Releasing Material of PEGlyated Ruthenium Carbonyl Complex
Source: Materials (Basel). 2022 May 18;15(10):3597. doi: 10.3390/ma15103597 (PMC9143562; doi:10.3390/ma15103597)
Supplement: Supplementary file 1 [file materials-15-03597-s001.zip › materials-1624474-supplementary.pdf]

Electronic Supplementary Information

Hydrophilic CO-releasing material of PEGlyated Ruthenium Carbonyl Complex

Xiao Zhang [1], Nan Guo [2], Shuhong Yang [1], Huma Khan [1], Weiqiang Zhang [1*]

[1] Key Laboratory of Applied Surface and Colloid Chemistry MOE, School of Chemistry and Chemical Engineering, Shaanxi Normal University, Xi'an 710062, China; zx@snnu.edu.cn(X.Z.); 917501881@qq.com(S.Y.); huma7662@gmail.com(H.K.)

[2] Northwest University, Xi'an 710127, China; 13991393343@163.com(N.G.)

* Correspondence: zwq@snnu.edu.cn; Tel.: +86-181-8243-8818 (W.Z.)

1. Preparation and Characterization of complexes 4-15..1

2. Crystal Data and Structure Refinement of 8, 13 and 14......................................4

3. CO-Release Measured by Standard Myoglobin Assay..7

4. Water-solubility Measured by the flask - shaking method..................................8

5. Copies of ^{1}H-NMR, ^{13}C-NMR and UV-Vis Spectra...9

6. Cell culture and Cytotoxicity studies..36

1. Preparation and Characterization of complexes 4-15

- $[Ru_2(CO)_4(\mu_2\text{-}\eta^2\text{-}O_2CCH_3)_2(\eta^1\text{-}NH_2CH_2C(=O)OCH_2CH_2OCH_3)_2]$ (**4**).

The thermolysis of $Ru_3(CO)_{12}$(200 mg, 0.31 mmol) and the acetic acid (15 mL) were heated at 120 °C in a Schlenk tube for 8h, afforded the pale yellow solution of $[Ru_2(O_2CCH_3)_2(CO)_4]_n$ (2) along with byproduct CO and H_2. The reaction mixture was cooled down 50 °C. mPEG amino acid esters (0.93 mmol) were neutralized by triethylamine in CH_2Cl_2:CH_3OH=1:1, and were added to the above reaction mixture via cannula filtration. The resultant mixture was stirred at 50°C for 2 h. The bright pale yellow solution was then briefly cooled, and the solvent was removed under vacuum. The crude powder product was recrystallized as yellow prismatic crystals. 120 mg (74 %). IR(CH_2Cl_2, cm^{-1}): Vco = 2025 vs, 1971 m, 1938 vs, V(coo) = 1743 m, Vacid = 1576 m, 1444 m. ^{1}H NMR(400 MHz, $CDCl_3$) δ 4.35(t, 2H, CH_2), 3.75(t, 2H, CH_2), 3.62(t, 2H, CH_2), 3.39(s, 3H, CH_3), 2.90(d, 2H, NH_2), 1.94(s, 3H, CH_3). ^{13}C NMR(101 MHz, $CDCl_3$) δ 203.81(CO), 184.24($COOCH_2$), 172.01(CH_3COO), 70.17(CH_2), 64.45(CH_2), 59.00(CH_2), 46.30(OCH_3), 23.34(CH_3). Anal. Calc. for $C_{18}H_{28}N_2O_{14}Ru_2$•0.5 CH_3OH: C 31.09, H 4.23, N 3.92. Found: C 31.17, H 3.82, N 3.18%.

- $[Ru_2(CO)_4(\mu_2\text{-}\eta^2\text{-}O_2CCH_3)_2(\eta^1\text{-}NH_2CH(CH_2Ph)C(=O)OCH_2CH_2OCH_3)_2]$ (**5**).

Yellow oil. Yield:158 mg(77 %). IR (CH_2Cl_2, cm^{-1}): Vco = 2025 vs, 1971 m, 1938 vs, V(coo) = 1739 m, Vacid = 1577 m, 1445 m. 1H NMR(400 MHz, $CDCl_3$) δ 7.24(dd, 3H, Ph), 7.20(d, 2H, Ph), 4.28(t, 2H, CH_2), 4.04(m, 1H, CH), 3.54(d, 2H, CH_2), 3.32(s, 3H, OCH_3), 3.21(m, 2H, CH_2), 3.03(t, 1H, NH_2), 2.57(t, 1H, NH_2), 1.83(s, 3H, CH_3). ^{13}C NMR (101 MHz, $CDCl_3$) δ 202.97(CO), 183.09(CH_2COO), 171.83(CH_3COO), 134.08(Ph), 128.48(Ph), 127.88(Ph), 126.43(Ph), 69.11(CH_2), 63.52(CH), 57.93(CH_2), 56.67(CH_3), 38.20(CH_2), 22.27(CH_3). Anal. Calc. for $C_{32}H_{40}N_2O_{14}Ru_2$: C 43.73, H 4.59, N 3.19. Found: C 44.04, H 4.65, N 3.170%.

- $[Ru_2(CO)_4(\mu_2\text{-}\eta^2\text{-}O_2CCH_3)_2(\eta^1\text{-}NH_2CH(CH_2Ph)C(=O)O(CH_2CH_2O)_2CH_3)_2]$ (**6**).

Yellow oil. Yield: 166 mg (74 %). IR (CH_2Cl_2, cm^{-1}): Vco = 2025 vs, 1971 m, 1938 vs, V(coo) = 1739 m, Vacid = 1576 m, 1445 m. H NMR (400 MHz, $CDCl_3$) δ 7.26(m, 3H, Ph), 7.19(m, 2H, Ph), 4.29(d, 2H, CH_2), 4.03(dd, 1H, CH), 3.65(t, 2H, CH_2), 3.61 - 3.54(m, 2H, CH_2), 3.51 - 3.45(m, 2H, CH_2), 3.31(s, 3H, OCH_3), 3.27 - 3.12(m, 2H, CH_2), 3.04(t, 1H, NH_2), 2.57(t, 1H, NH_2), 1.83(s, 3H, CH_3).^{13}C NMR (101 MHz, $CDCl_3$) δ 203.00(CO), 183.09(CH_2COO), 171.79(CH_3COO), 134.12(Ph), 128.49(Ph), 127.88(Ph), 126.42(Ph), 70.86(CH_2), 69.48(CH), 67.80(CH_2), 63.61(CH_2), 58.06(CH_2), 56.72(OCH_3), 38.16(CH_2), 22.28(CH_3). Anal. Calc. for $C_{36}H_{48}N_2O_{16}Ru_2$•0.5 CH_2C_{l2}: C 43.43, H 4.89, N 2.78. Found: C 42.90, H 4.75, N 3.0%.

- $[Ru_2(CO)_4(\mu_2\text{-}\eta^2\text{-}O_2CCH_3)_2(\eta^1\text{-}NH_2CH(CH_2Ph)C(=O)O(CH_2CH_2O)_3CH_3)_2]$ (**7**).

Yellow oil. Yield: 128 mg (52 %). IR (CH_2Cl_2, cm^{-1}): Vco = 2025 vs, 1971 m, 1938 vs, V(coo) = 1740 m, Vacid = 1575 m, 1444 m. ^{1}H NMR (400 MHz, $CDCl_3$) δ 7.25(dd, 3H, Ph), 7.19(m, 2H, Ph), 4.28(dd, 2H, CH_2), 4.06 - 3.98(m, 1H, CH), 3.65 (s, 2H, CH_2), 3.62 - 3.54(m, 6H, CH_2), 3.49 - 3.44(m, 2H, CH_2), 3.30(s, 3H, OCH_3), 3.26 - 3.12(m, 2H, CH_2), 3.04(t, 1H, NH_2), 2.58(t, 1H, NH_2), 1.83(s, 3H, CH_3).13C NMR (101 MHz, $CDCl_3$) δ 203.01(CO), 183.09(CH_2COO), 171.80(CH_3COO), 134.12(Ph), 128.49(Ph), 127.87(Ph), 126.42(Ph), 70.89(CH_2), 69.57(CH), 67.76(CH_2), 63.65(CH_2), 58.01(CH_2), 56.71(OCH_3), 38.13(CH_2), 22.28(CH_3). Anal. Calc. for $C_{40}H_{56}N_2O_{18}Ru_2$•1 CH_2C_{l2}: C 43.20, H 5.13, N 2.46. Found: C 43.37, H 5.21, N 2.71%.

- $[Ru_2(CO)_4(\mu_2\text{-}\eta^2\text{-}O_2CC_6H_5)_2(\eta^1\text{-}NH_2CH_2C(=O)OCH_2CH_2OCH_3)_2]$ (**8**).

A amount of $Ru_3(CO)_{12}$ (200 mg, 0.31 mmol) and the carboxylic acid (0.93 mmol) in dry Toluene (5 mL) were heated at 120 oC in a Schenk tube for 8 h. Then, add three equivalents of the axial ligand (0.93 mmol) and triethylamine neutralized in CH_2Cl_2 and CH_3OH mixed solvent, and filtered to the above reaction solution. The reaction stirring at 50 °C for 2 h. The bright yellow solution was cooled, and the solvent was removed by vacuum pump, affords deep yellow crystalline powder. 264 mg (yield 69%). IR (CH_2Cl_2, cm^{-1}): Vco = 2026

vs, 1973 m, 1941 vs, V(coo) = 1743 m, Vacid = 1597 m, 1558 m; ^{1}H NMR(400 MHz, $CDCl_3$): δ(ppm) 7.89(d, , 2H, Ph), 7.44(t, 1H, Ph), 7.33(t, 2H, Ph), 4.42(t, 2H, CH_2), 3.96(t , 2H, CH_2), 3.67(t, 2H, CH_2), 3.42(s, 3H, OCH_3), 3.16(s, 2H, NH_2); ^{13}C NMR(101 MHz, $CDCl_3$): 202.66(CO), 177.67(CO_2CH_2), 170.97($PhCO_2$), 130.81(Ph), 128.55(Ph), 128.01(Ph), 126.91(Ph), 69.18(CH_2), 63.62(CH_2), 58.05(CO_2CH_3), 45.36(CH_3). Anal. Calc. for $C_{28}H_{32}N_2O_{14}Ru_2$: C 40.88, H 3.92; N 3.41. Found: C 40.44, H 3.96, N 3.21%.

- $[Ru_2(CO)_4(\mu_2\text{-}\eta^2\text{-}O_2C\text{-}o\text{-}CH_3C_6H_5)_2(\eta^1\text{-}NH_2CH_2C(=O)OCH_2CH_2OCH_3)_2]$ (**9**).

Deep yellow wax solid. Yield: 275 mg (72%). IR (CH_2Cl_2, cm^{-1}):Vco = 2026 vs, 1973 m, 1941 vs, V(coo) = 1743 m, Vacid = 1597 m, 1556 m; ^{1}H NMR(400 MHz, $CDCl_3$): δ(ppm) 7.63(d, 1H, Ph), 7.29(t, 1H, Ph), 7.16(t, 2H, Ph), 4.42-4.25(m, 2H, CH_2), 3.87(t, 2H, CH_2), 3.67-3.56(m, 2H, CH_2), 3.39(s, 3H, OCH_3), 3.05(t, 2H, NH_2), 2.41(s, 3H, $PhCH_3$); ^{13}C NMR(101 MHz, $CDCl_3$): 202.69(CO), 180.35(CO_2CH_2) , 170.76($PhCO_2$), 136.26(Ph), 133.26(Ph), 130.03(Ph), 129.47(Ph), 128.58(Ph), 124.50 (Ph), 69.10(CH_2), 63.53(CH_2), 57.99(CO_2CH_3), 45.77(CH_3), 20.65($PhCH_3$). Anal. Calc. for $C_{30}H_{36}N_2O_{14}Ru_2$: C 42.35, H 4.27, N 3.29. Found: C 42.28, H 4.31, N 3.17%.

- $[Ru_2(CO)_4(\mu_2\text{-}\eta^2\text{-}O_2C\text{-}m\text{-}CH_3C_6H_5)_2(\eta^1\text{-}NH_2CH_2C(=O)OCH_2CH_2OCH_3)_2]$ (**10**).

Deep yellow crystalline powder. Yield: 283 mg(74 %). IR (CH_2Cl_2, cm^{-1}):Vco = 2026 vs, 1973 m, 1940 vs, V(coo) = 1744 m, Vacid = 1560 m; ^{1}H NMR(400 MHz, $CDCl_3$): δ(ppm) 7.69(d, 2H, Ph), 7.25 - 7.09(m, 2H, Ph), 4.50 - 4.36(m, 2H, CH_2), 3.97(t, 2H, CH_2), 3.71 - 3.60(m, 2H, CH_2), 3.42(s, 3H, OCH_3), 3.16(s, 2H, NH_2), 2.35 (s, 3H, $PhCH_3$); ^{13}C NMR(101 MHz, $CDCl_3$) 202.68(CO), 177.83(CO_2CH_2), 171.00 ($PhCO_2$), 136.56(Ph), 131.99(Ph), 131.52(Ph) , 129.11(Ph) , 126.80(Ph), 125.74(Ph), 69.18(CH_2), 63.60(CH_2), 58.03(CO_2CH_3), 45.50(CH_3), 20.25($PhCH_3$). Anal. Calc. for $C_{30}H_{36}N_2O_{14}Ru_2$•0.5 CH_2Cl_2: C 41.01, H 4.18, N 3.14. Found: C 41.5, H 4.13, N 3.06%.

- $[Ru_2(CO)_4(\mu_2\text{-}\eta^2\text{-}O_2C\text{-}p\text{-}CH_3C_6H_5)_2(\eta^1\text{-}NH_2CH_2C(=O)OCH_2CH_2OCH_3)_2]$ (**11**).

Deep yellow crystalline powder. Yield: 311 mg(79 %). IR (CH_2Cl_2, cm^{-1}):Vco = 2025 vs, 1972 m, 1940 vs, V(coo) = 1743 m, Vacid = 1593 m, 1552 m; ^{1}H NMR(400 MHz, $CDCl_3$): δ(ppm) 7.76(d, 2H, Ph), 7.11(d, 2H, Ph), 4.50 - 4.31(m, 2H, CH_2), 3.95 (t, 2H, CH_2), 3.67(dd, 2H, CH_2), 3.42(s, 3H, OCH_3), 3.13(t, 2H, NH_2), 2.34(s, 3H, $PhCH_3$); ^{13}C NMR (101 MHz, $CDCl_3$) δ 202.75(CO), 177.70(CO_2CH_2), 171.01($PhCO_2$), 141.15(Ph), 129.42(Ph), 128.57(Ph), 127.55(Ph), 69.19(CH_2), 63.58 (CH_2), 58.04(CH_2), 45.35(CH_3), 20.48($PhCH_3$). Anal. Calc. for $C_{30}H_{36}N_2O_{14}Ru_2$: C 42.35; H 4.27; N 3.29. Found: C 41.9, H 4.26, N 3.19%.

- $[Ru_2(CO)_4(\mu_2\text{-}\eta^2\text{-}O_2C\text{-}o\text{-}OCH_3C_6H_5)_2(\eta^1\text{-}NH_2CH_2C(=O)OCH_2CH_2OCH_3)_2]$ (**12**).

Yellow crystalline powder. Yield: 260 mg (63 %). IR (CH_2Cl_2, cm^{-1}): Vco = 2025 vs, 1971 m, 1939 vs, V(coo) = 1742 m, Vacid = 1604 m, 1559 m; ^{1}H NMR(400 MHz, $CDCl_3$): δ(ppm) 7.64(d, 1H, Ph), 7.36 – 7.32(m, 1H, Ph), 6.90 – 6.86(m, 2H, CH_2), 4.35 – 4.33(m, 2H, CH_2), 3.95 – 3.92(m, 2H, CH_2), 3.74(s, 3H, $PhOCH_3$), 3.62 – 3.60(m, 2H, CH_2), 3.38(s, 3H, OCH_3), 3.12 – 3.08(t, 2H, NH_2). ^{13}C NMR (101 MHz, $CDCl_3$) δ 203.93 (CO), 178.86 (CO_2CH_2), 172.35 ($PhCO_2$), 158.21 (Ph), 132.03(Ph), 131.27 (Ph), 123.91(Ph), 119.88 (Ph), 111.91 (Ph), 70.20 (CH_2), 64.34(CH_2), 58.97 ($PhOCH_3$), 55.55 (CH_2), 46.09 (CH_3). Anal. Calc. for $C_{30}H_{36}N_2O_{16}Ru_2$: C 40.82; H 4.11; N 3.17. Found: C 40.73, H 3.75, N 2.95%.

- $[Ru_2(CO)_4(\mu_2\text{-}\eta^2\text{-}O_2C\text{-}p\text{-}ClC_6H_5)_2(\eta^1\text{-}NH_2CH_2C(=O)OCH_2CH_2OCH_3)_2]$ (**13**).

Yellow crystalline powder. Yield: 300 mg(72 %). IR (CH_2Cl_2, cm^{-1}):Vco = 2028 vs, 1974 m, 1939 vs, V(coo) = 1743 m, Vacid = 1596 m, 1555 m; ^{1}H NMR (400 MHz, $CDCl_3$): δ(ppm) 7.80(d, 2H, Ph), 7.29(d, 2H, Ph), 4.48 - 4.34(m, 2H, CH_2), 3.93(t, 2H, CH_2), 3.72 - 3.61(m, 2H, CH_2), 3.42(s, 3H, OCH_3), 3.13(s, 2H, NH_2); ^{13}C NMR (101 MHz, $CDCl_3$) δ 202.33(CO), 176.79(CO_2CH_2), 170.87($PhCO_2$), 137.20(Ph), 130.29(Ph), 129.98(Ph), 127.19(Ph), 69.15(CH_2), 63.68(CH_2), 58.05(CH_2), 45.41(CH_3). Anal. Calc. for $C_{28}H_{30}Cl_2N_2O_{14}Ru_2$•0.5 CH_2Cl_2: C 36.65, H 3.35, N 3.00. Found: C 36.79, H 3.65, N 2.81%.

- $[Ru_2(CO)_4(\mu_2\text{-}\eta^2\text{-}O_2C\text{-}C_{10}H_7)_2(\eta^1\text{-}NH_2CH_2C(=O)OCH_2CH_2OCH_3)_2]$ (**14**).

Yellow crystalline powder. Yield: 284 mg (66 %). IR (CH_2Cl_2, cm^{-1}):Vco = 2027 vs, 1974 m, 1943 vs, V(coo) = 1743 m, Vacid = 1600 w, 1559 m; ^1H NMR (400 MHz, $CDCl_3$): δ(ppm) 8.42(d, 1H, $C_{10}H_7$), 7.91 - 7.81(m, 3H, $C_{10}H_7$), 7.42(dd, 3H, $C_{10}H_7$), 4.35 - 4.23 (m, 2H, CH_2), 3.88(t, 2H, CH_2), 3.58 - 3.49(m, 2H, CH_2), 3.33(s, 3H, OCH_3), 3.14(s, 2H, NH_2); ^{13}C NMR (101 MHz, $CDCl_3$) δ 202.69(CO), 180.52(CO_2CH_2), 170.68($C_{10}H_7CO_2$), 132.64($C_{10}H_7$), 131.61($C_{10}H_7$), 130.23($C_{10}H_7$), 129.39($C_{10}H_7$), 128.01($C_{10}H_7$), 127.31($C_{10}H_7$), 126.93($C_{10}H_7$), 125.87($C_{10}H_7$), 124.94($C_{10}H_7$), 123.64($C_{10}H_7$), 69.05(CH_2), 63.46(CH_2), 57.94(CH_2), 45.52(CH_3). Anal. Calc. for $C_{36}H_{36}N_2O_{14}Ru_2$: C 46.85; H 3.93; N 3.04. Found: C 47.11, H 4.11, N 2.96%.

- $[Ru_2(CO)_4(\mu_2\text{-}\eta^2\text{-}O_2C\text{-}C_{14}H_9)_2(\eta^1\text{-}NH_2CH_2C(=O)OCH_2CH_2OCH_3)_2]$ (**15**).

Yellow crystalline powder. Yield: 291 mg (61 %). IR (CH_2Cl_2, cm^{-1}):Vco = 2028 vs, 1976 m, 1944 vs, V(coo) = 1744 m, Vacid = 1562 m; ^1H NMR (400 MHz, $CDCl_3$): δ(ppm) 8.50(s, 1H, $C_{14}H_9$), 8.08(d, 2H, $C_{14}H_9$), 8.00(d, 2H, $C_{14}H_9$), 7.43 - 7.36(m, 2H, $C_{14}H_9$), 7.19(t, 2H, $C_{14}H_9$), 4.09 - 4.01(m, 2H, CH_2), 3.73(t, 2H, CH_2), 3.36 - 3.31(m, 2H, CH_2), 3.21(s, 3H, OCH_3), 3.07(t, 2H, NH_2); ^{13}C NMR (101 MHz, $CDCl_3$) δ 202.77(CO), 182.42(CO_2CH_2), 170.27($C_{14}H_9CO_2$), 130.90($C_{14}H_9$), 130.11($C_{14}H_9$), 128.01($C_{14}H_9$), 127.40($C_{14}H_9$), 126.96($C_{14}H_9$), 126.69($C_{14}H_9$), 125.54($C_{14}H_9$), 124.86($C_{14}H_9$), 124.35($C_{14}H_9$), 68.85(CH_2), 63.26(CH_2), 57.79(CH_2), 45.26(CH_3).Anal. Calc. for $C_{44}H_{40}N_2O_{14}Ru_2$: C 51.66; H 3.94; N 2.74. Found: C 51.34, H 4.37, N 2.66%.

2. Crystal Data and Structure Refinement of 8, 13 and 14

Single-crystal X-ray diffraction data were collected on a Gemini E CCD diffractometer with a CCD bidimensional equipped with graphite monochromated MoKα radiation (λ = 0.71073 Å), at 296K(**8, 13, 14**). The crystal was fixed at the tip of a glass fiber loop, and after the loop was mounted on the goniometer head, it was optically centered. Data integration and reduction were performed using SaintPlus 6.01.[1] Absorption correction was performed by multi-scan method implemented in SADABS.[2] Space groups were determined using XPREP implemented in APEX2. The structures were solved by the direct method using Olex2.[3] The full-matrix least-squares refinement on F^2 included atomic coordinates and anisotropic thermal parameters for all non-hydrogen atoms. Hydrogen atoms were located at geometrically calculated positions to their carrier atoms and refined with isotropic thermal parameters included in the final stage of the refinement. Crystal data and refinement conditions are listed in Table S1 in the Supporting Information. Crystallographic data for the structures have been deposited with the Cambridge Crystallographic Data Centre. Copies of the data can be obtained free of charge on quoting the depository numbers 1037349 (**8**), 1061086 (**13**), and 1061038 (**14**). All the crystals were obtained in dichloromethane and petroleum ether mixed solvents at -20℃. These data can be obtained free of charge from the Cambridge Crystallographic Data Centre via www.ccdc.cam.ac.uk/data_request/cif.

Table S1. Data Collection and Structural Refinements Details for Single-Crystal X-ray Diffraction Studies of Complexes 8, 13 and 14

	**8**	**13**	**14**
Empirical formula	$C_{28}H_{32}N_2O_{14}Ru_2$	$C_{28}H_{32}Cl_2N_2O_{15}Ru_2$	$C_{36}H_{38}N_2O_{15}Ru_2$
Formula weight	822.70	909.60	940.82
T(K)	296(2)	296(2)	296(2)
Wavelength (Å)	0.71073	0.71073	0.71073
Crystal system	Monoclinic	Monoclinic	Monoclinic
Space group	C2/c	C2/c	C2/c
a (Å)	29.592(6)	25.674(5)	30.5521(7)
b (Å)	14.559(3)	13.956(3)	14.4710(3)
c (Å)	14.559(3)	21.387(4)	19.9839(4)
α (°)	90	90	90
β (°)	92.41(3)	106.65(3)	99.875
γ (°)	90	90	90
V(Å^3)	7772(3)	7342(3)	8704.4(3)
Z	8	8	8
Calculated density	1.406 Mg/m^3	1.646 Mg/m^3	1.436 Mg/m^3
Absorption coefficient	0.835 mm^-1	1.036 mm^-1	0.758 mm^-1
F(000)	3312	3648	3808
Crystal size(mm^3)	0.14 x 0.13 x 0.12 mm	0.14 x 0.13 x 0.11 mm	0.16 x 0.14 x 0.10 mm
θ range for data collection(deg)	3.30 to 26.00	3.08 to 25.99	3.22 to 26.00

Limiting indices	-36<=h<=36, -17<=k<=17, -22<=l<=21	-31<=h<=31, -17<=k<=17, -26<=l<=26	-37<=h<=37, -17<=k<=17, -24<=l<=23
Reflections collected / unique	39683 / 7626 [R(int) = 0.0472]	37491 / 7175 [R(int) = 0.0372]	45495 / 8524 [R(int) = 0.0423]
Completeness to θ = 26.00	99.7 %	99.7 %	99.6 %
Absorption correction	Semi-empirical from equivalents	Semi-empirical from equivalents	Semi-empirical from equivalents
Max. and min.	0.9064 and 0.8920	0.8946 and 0.8686	0.9281 and 0.8884
Refinement method	Full-matrix least-squares on F^2	Full-matrix least-squares on F^2	Full-matrix least-squares on F^2
Data / restraints / parameters	7626 / 5 / 416	7175 / 34 / 443	8524 / 0 / 498
GOF on F^2	1.195	1.130	1.057
Final R indices [I>2δ(I)]	R1 = 0.0596, wR2 = 0.1386	R1 = 0.0453, wR2 = 0.1169	R1 = 0.0443, wR2 = 0.1212
R indices (all data)	R1 = 0.0931, wR2 = 0.1744	R1 = 0.0620, wR2 = 0.1336	R1 = 0.0691, wR2 = 0.1499
Largest diff. peak and hole(e.$Å^{-3}$)	1.569 and -0.740	1.938 and -0.870	0.933 and -0.493

Table S2. **Selected Bond Lengths (Å) for Complexes 8, 13 and 14**

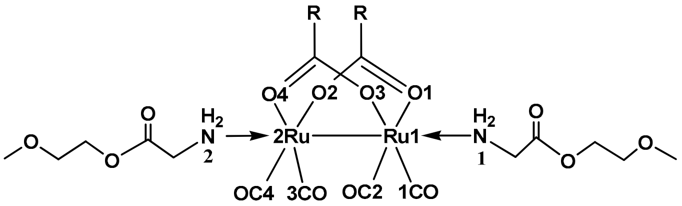

compounds	**8**	**13·H_2O**	**14**
Ru(1)-C(1)	1.830(4)	1.825(5)	1.822(7)
Ru(1)-C(2)	1.835(4)	1.832(5)	1.833(8)
Ru(1)-O(2)	2.120(2)	2.116(3)	2.113(4)
Ru(1)-O(1)	2.126(2)	2.121(3)	2.114(5)
Ru(1)-N(1)	2.248(3)	2.261(3)	2.270(6)
Ru(1)-Ru(2)	2.6760(3)	2.6742(5)	2.6754(7)
Ru(2)-C(3)	1.819(4)	1.821(4)	1.833(8)
Ru(2)-C(4)	1.834(4)	1.833(5)	1.843(9)
Ru(2)-O(3)	2.129(2)	2.119(2)	2.123(4)
Ru(2)-O(4)	2.141(2)	2.120(2)	2.128(6)
Ru(2)-N(2)	2.263(2)	2.264(3)	2.261(6)
C(1)-Ru(1)-C(2)	89.69(16)	89.75(19)	87.4(3)

C(4)-Ru(2)-O(3)	177.75(13)	178.22(14)	178.7(3)
O(3)-Ru(2)-O(4)	84.28(9)	85.29(10)	85.2(2)
N(1)-Ru(1)-Ru(2)	163.44(8)	164.36(9)	162.86(19)
O(1)-Ru(1)-Ru(2)	85.16(6)	84.65(7)	83.99(14)
O(2)-Ru(1)-N(1)	84.92(9)	86.24(11)	83.5(2)
C(1)-Ru(1)-N(1)	95.91(12)	95.00(16)	98.2(3)

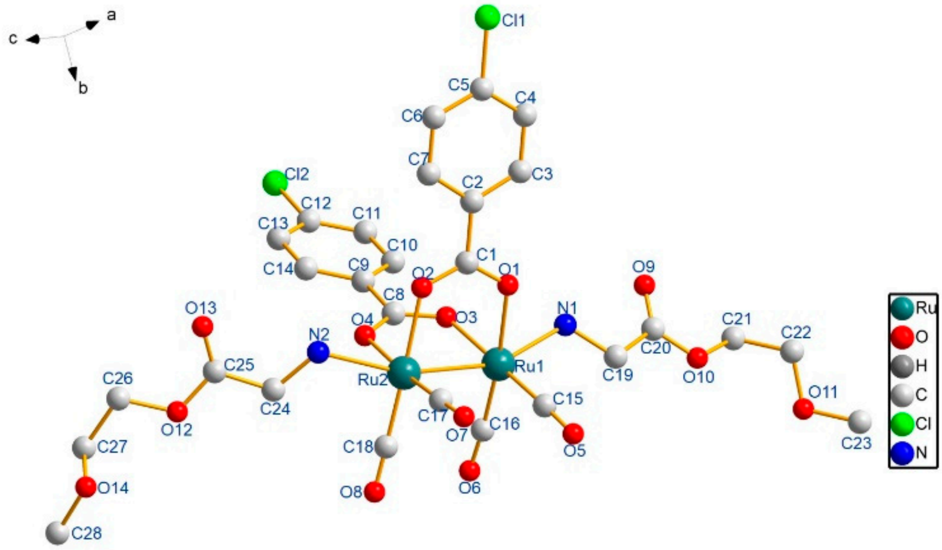

Figure S1 Molecular structure of complex **13** (H atoms are omitted for clarity).

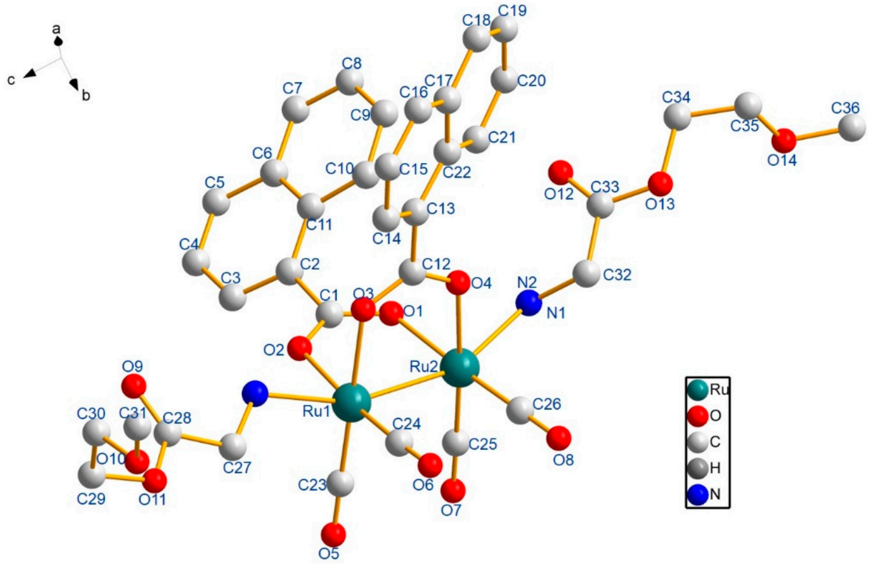

Figure S2 Molecular structure of complex **14** (H atoms are omitted for clarity).

3. CO-Release Measured by Standard Myoglobin Assay

The release of CO from the metal carbonyl compounds was studied by measuring the conversion of deoxy-myoglobin (deoxy-Mb) to carboxy-myoglobin (Mb-CO). The amount of Mb-CO formed was quantified by measuring the absorbance at 540 nm. A stock solution of myoglobin (lyophilised horse heart) (66 μM final concentration) was prepared fresh by dissolving the protein in phosphate buffered saline (PBS) (0.01M, pH = 7.4). Sodium dithionite (0.1%) was added to convert the myoglobin stock to deoxy-Mb. A 2 mL quantity of this was measured to obtain a deoxy-Mb spectrum and then bubbled with CO to get a Mb-CO spectrum. CO-RMs were dissolved in an appropriate solvent (DMSO or EtOH) (4, 8, 12 μM) and added to deoxy-Mb in the cuvette (to give a final CO-RM concentration of 20, 40, 60 μM), mixed using a pipette and then overlaid with 500 μL light mineral oil to prevent CO escaping or the myoglobin being oxygenated.The cuvette was loaded in the chamber of UV spectrometer with an LED (365nm, 2.5W) on the top. The first spectrum (t=0s) was record without UV irradiation. The CO release were then initiated by UV irradiation. Each UV spectrum was record when LED turn off during scanning for a short time intervals. This is the standard procedure; other experiments have been undertaken using different concentrations of myoglobin and different concentrations of DMSO. The maximal absorption peak of deoxy-Mb at 560 nm is converted to the two maximal absorption peaks of Mb-CO at 540 and 578 nm. The concentration of myoglobin in the stock solution was calculated from the maximal absorption peak of the Mb-CO solution at 540 nm (Equation 1).

$$\textbf{Mb-COmax} = (\textbf{OD540} / \varepsilon) \times 1000$$

Equation 1. Equation for calculating total myoglobin concentration in a saturated solution of Mb-CO; ε = extinction coefficient of Mb-CO=15.4 $mM^{-1}cm^{-1}$, OD540 = absorbance of Mb-CO solution at 540 nm.

Intermediate concentrations of Mb-CO are calculated from the OD540. A new extinction coefficient (ε2) must be calculated to take into account the change in absorbance at 540 nm (ΔOD540). To aid in the accuracy of this calculation, another wavelength is used as a constant reference point. The deoxy-Mb and Mb-CO spectra share four isosbestic (ODiso) points (510, 550, 570, 585 nm). The value at 510 nm (ODiso510) was used in this set of experiments. The new extinction coefficient was calculated (Equation 2).

$$\varepsilon 2 = (\Delta \textbf{OD540} - \Delta \textbf{ODiso510} \times 1000) / \textbf{Mb-COmax}$$

Equation 2. Equation needed to calculate unknown Mb-CO extinction coefficient. ΔODiso510=change in absorbance at the isosbestic point, ΔOD540=change in absorbance at 540 nm, Mb-COmax=maximum concentration of myoglobin. ε2 = new extinction coefficient.

From the new extinction coefficient and the change in absorbance at 540 and 510 nm will give the concentration of myoglobin in any unknown sample. (Equation 3)

$$\textbf{Mb-CO} = 1000 \times (\Delta \textbf{OD540} - \Delta \textbf{ODiso510}) / \varepsilon 2$$

Equation 3. Equation to calculate the Mb-CO concentration in samples. ΔOD540 = change in absorbance at 540 nm, ΔODiso510 = change in absorbance at the isosbestic point, ε2 = calculated absorption coefficient. The resulting curves for the formation of Mb-CO verus time were fitted using non-linear regression routines in SigmaPlot, resulting R^2 values were typically greater than 0.99. Half lives for CO-release were determined by extrapolating the equations generation from the non-linear regression to 30 μM, 20 μM and 10 μM Mb-CO for initial CO-RM concentrations of 60 μM, 40 μM and 20 μM respectively. The same method was employed for CORM which exhibited slow

release for Mb-CO concentrations of 15μM, 10μM and 5μM respectively. In the case of complexes 3a-3g significant baseline drift was observed in the Mb-CO assay which was corrected with the aid of a non-linear regression algorithm being applied to the isosbestic points [3].

4. Water-solubility Measured by the flask - shaking method

The lipophilicity of complexes was measured by the flask- shaking method where n-octanol and ultrapure water were used as the organic and aqueous phase respectively. First, make the pre saturated of oil-water two phase. Then the tested complex was dissolved in the isolated organic phase at a concentration of 1mg/mL, take 2 mL of liquld and 8mL aqueous phase was joined, the mixture was swirled for 12h at room temperature. The solution was then centrifuged, and the amounts of complex in organic layers were determined by UV-vis. Drawing standard curve and determination of oil-water partition coefficient. After the nonlinear fitting to the standard curve, the correlation coefficient $R^2>0.98$, indicating absorbance and the concentration has a good linear relationship, so it can take advantage of the absorbance to determine concentration of the tested complex.

5. Copies of ^{1}H-NMR, ^{13}C-NMR and UV-vis spectra

5.1 NMR spectra of Complex 4-7

$[Ru_2(CO)_4(\mu^2\text{-}\eta^2\text{-}O_2CCH_3)_2(\eta^1\text{-}NH_2CH_2C(=O)OCH_2CH_2OCH_3)_2]$ (4).

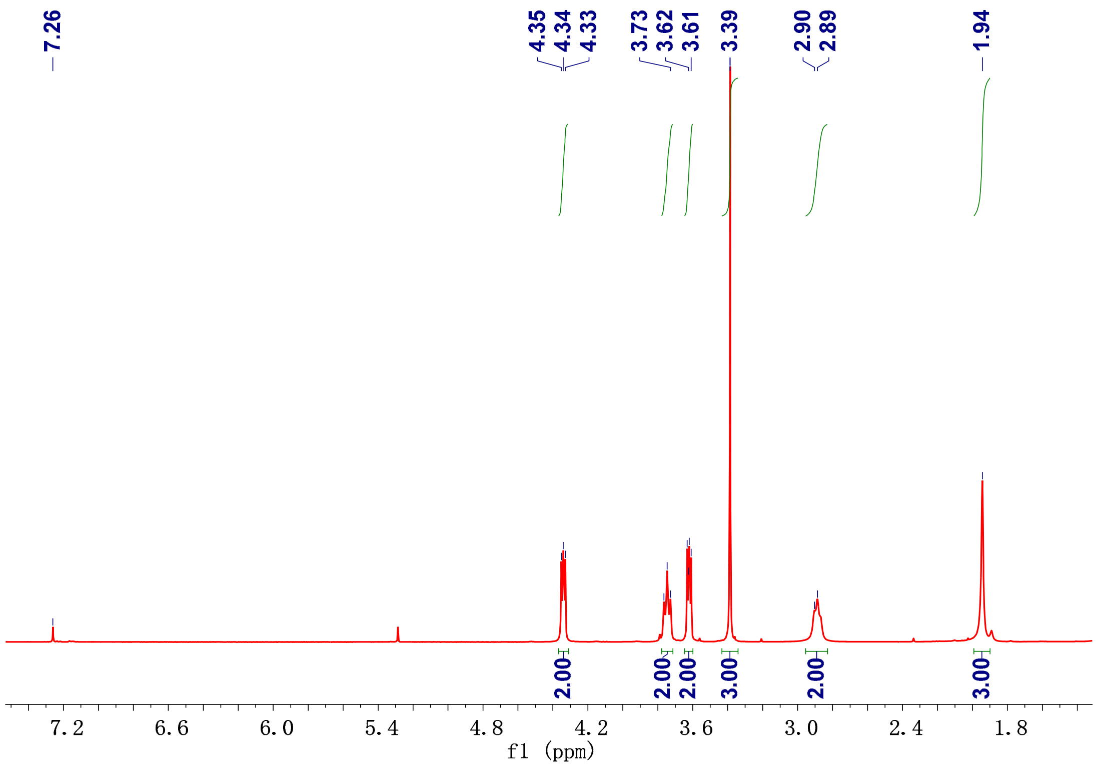

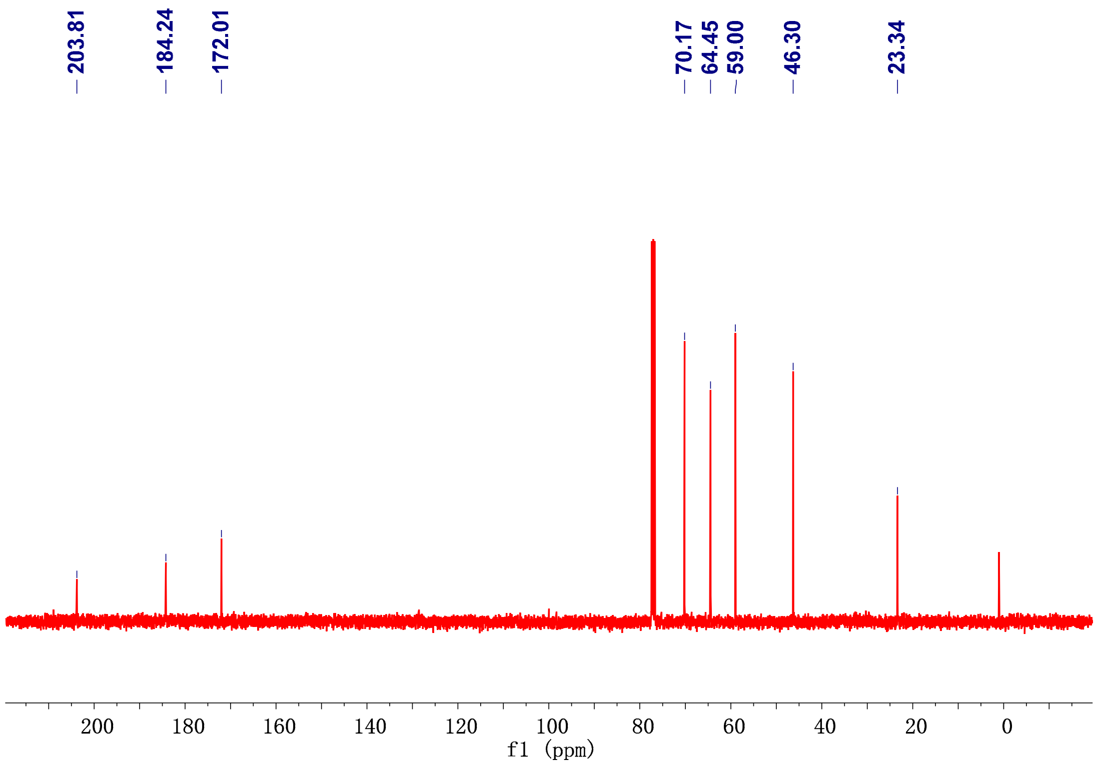

[Ru$_2$(CO)$_4$(μ^2-η^2-O$_2$CCH$_3$)$_2$ (η^1-NH$_2$CH(CH$_2$Ph)C(=O)OCH$_2$CH$_2$OCH$_3$)$_2$] (5)

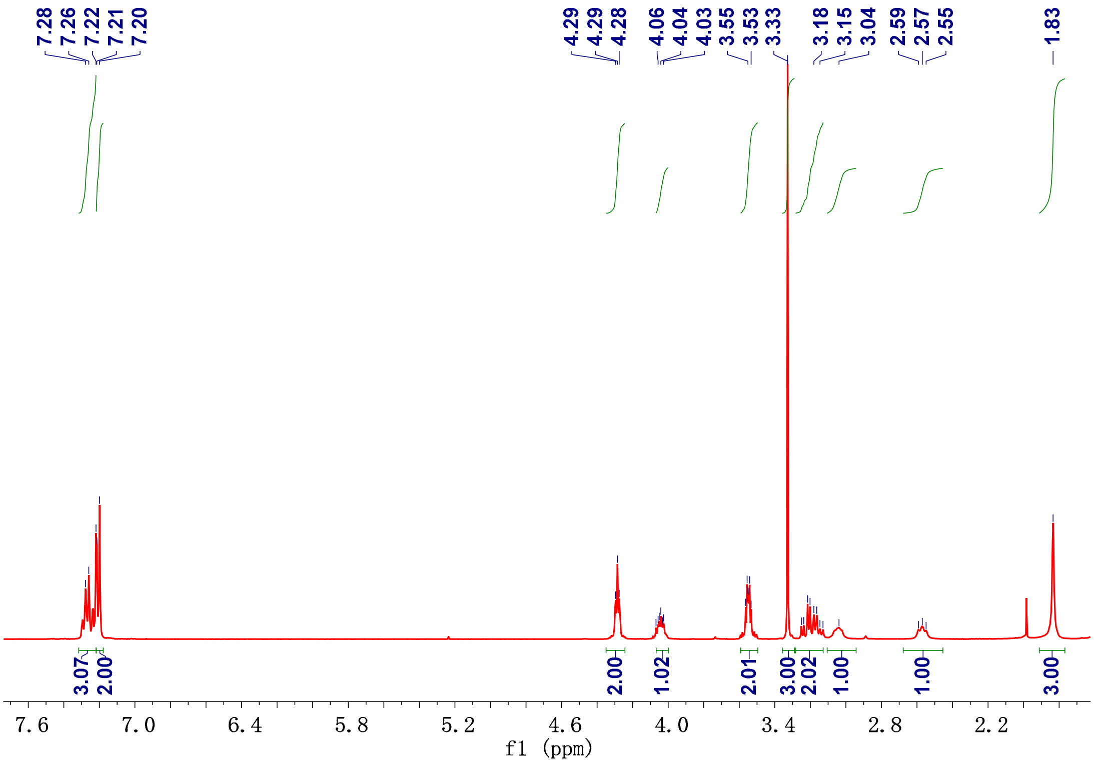

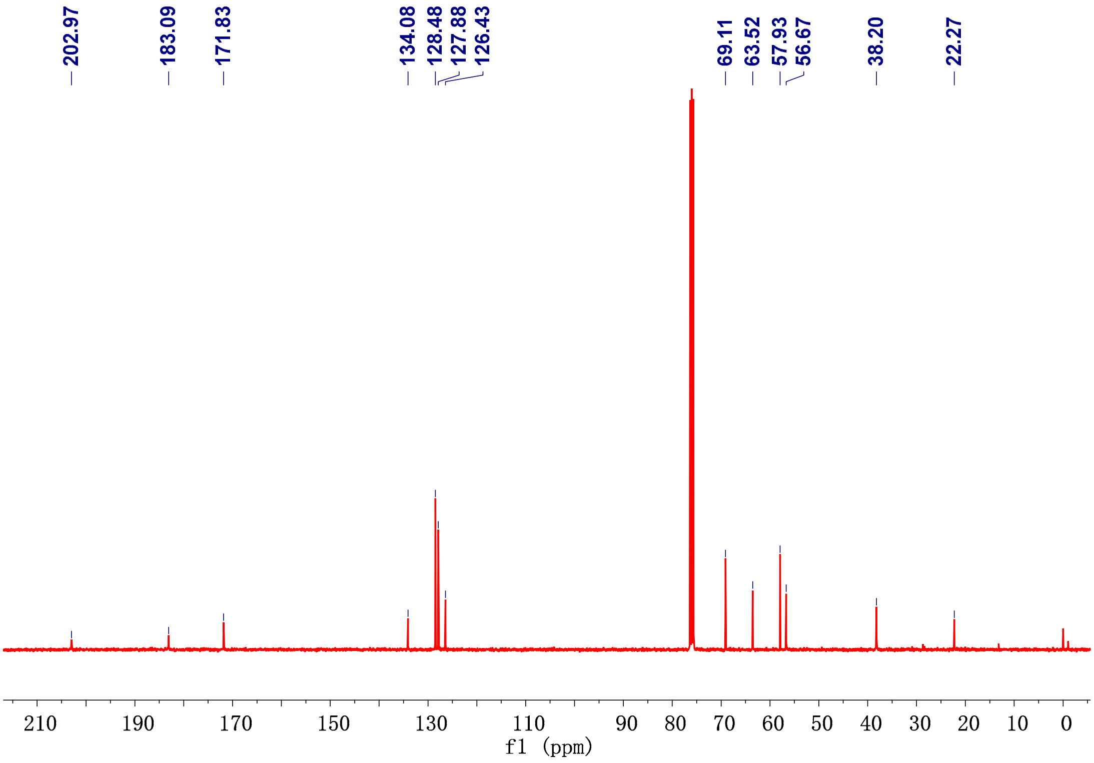

$[Ru_2(CO)_4(\mu^2\text{-}\eta^2\text{-}O_2CCH_3)_2\ (\eta^1\text{-}NH_2CH(CH_2Ph)C(=O)O(CH_2CH_2O)_2CH_3)_2]$ (6)

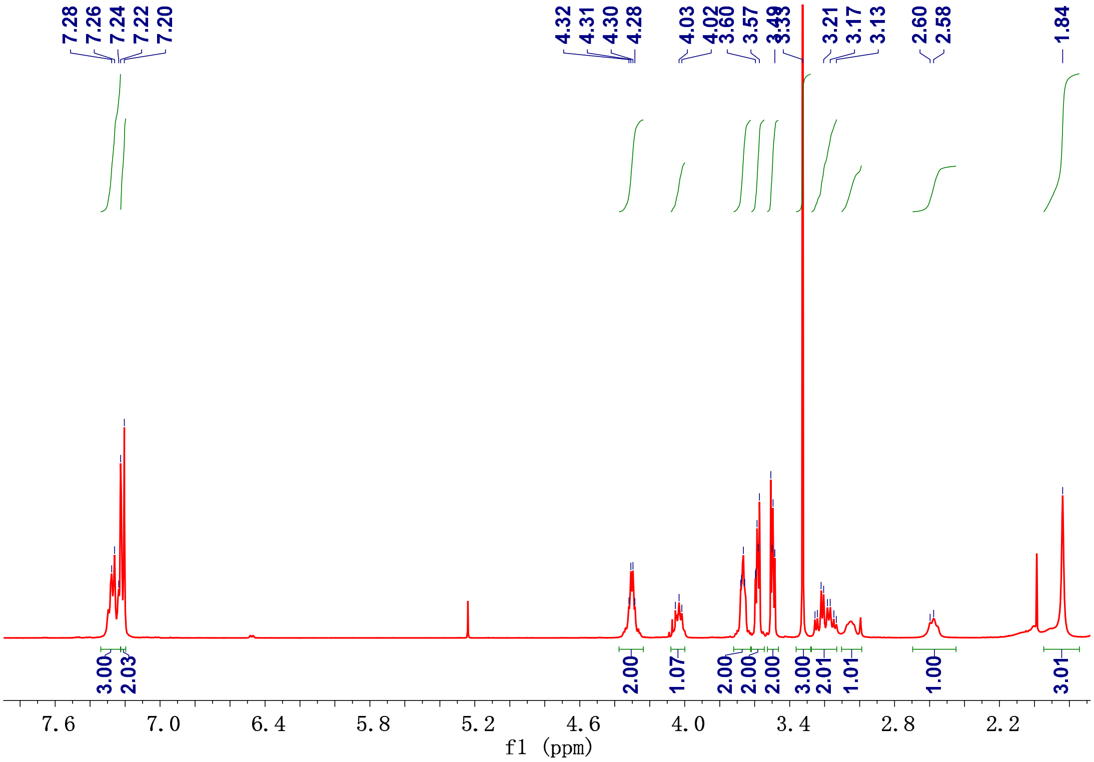

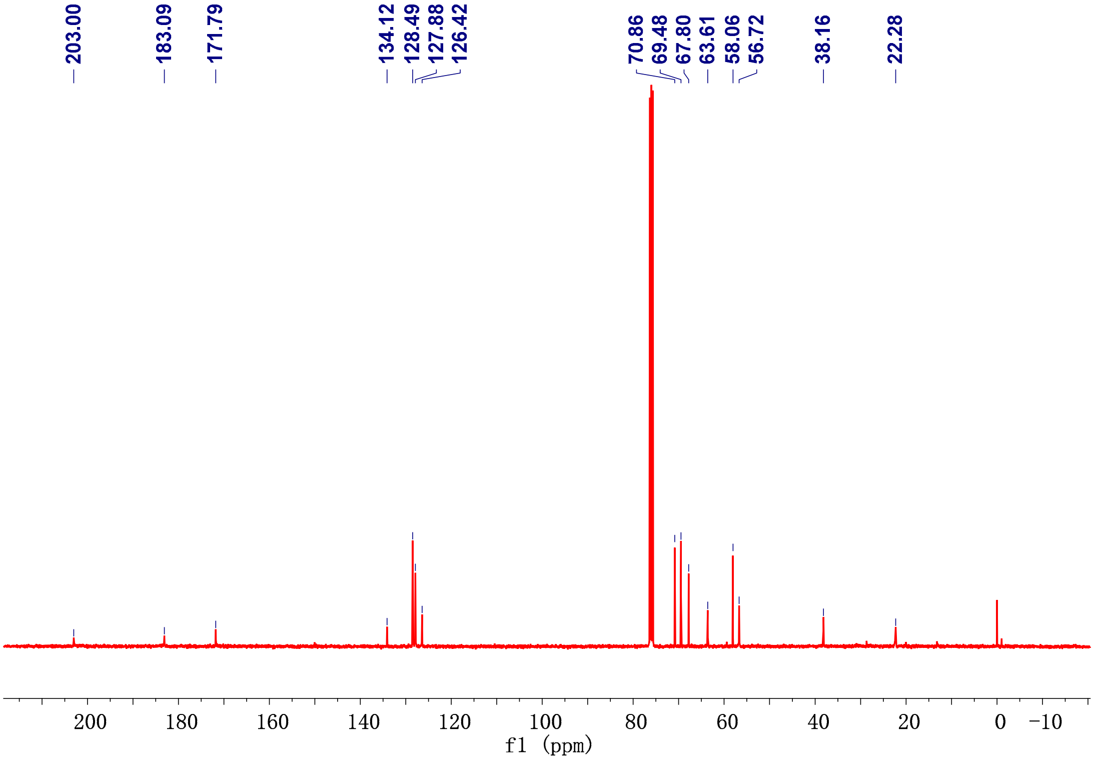

[$Ru_2(CO)_4(\mu^2$-η^2-$O_2CCH_3)_2$ (η^1-$NH_2CH(CH_2Ph)C(=O)O(CH_2CH_2O)_3CH_3)_2$] (7)

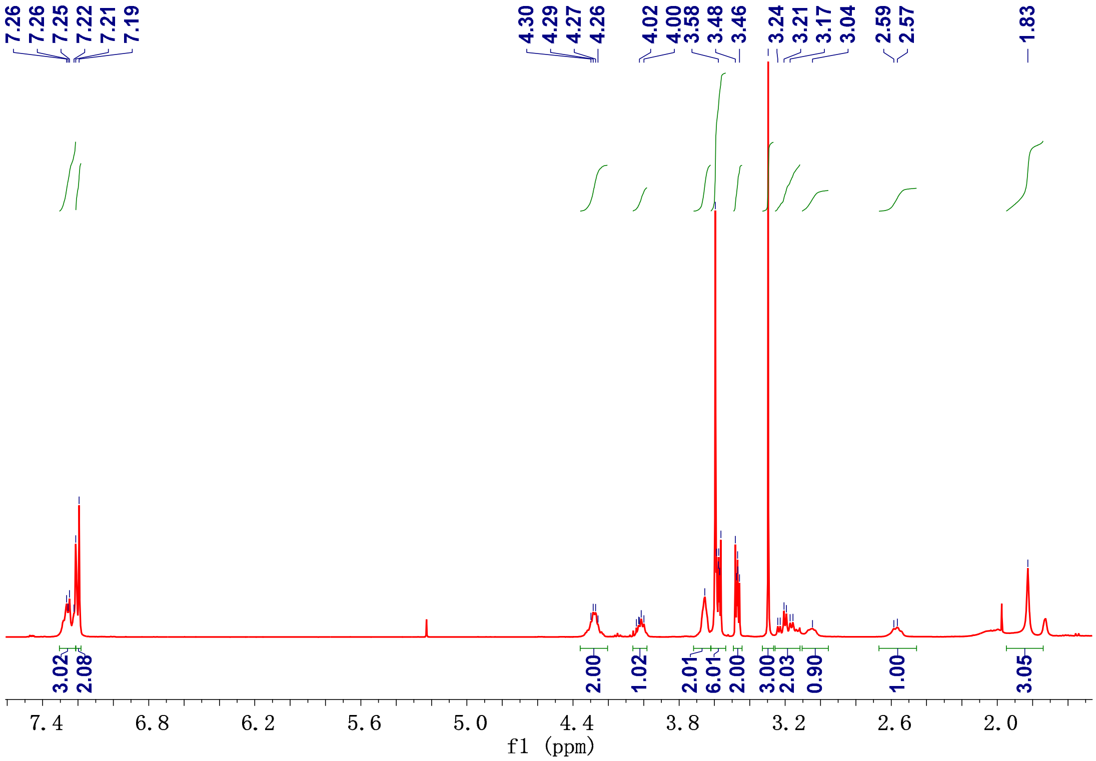

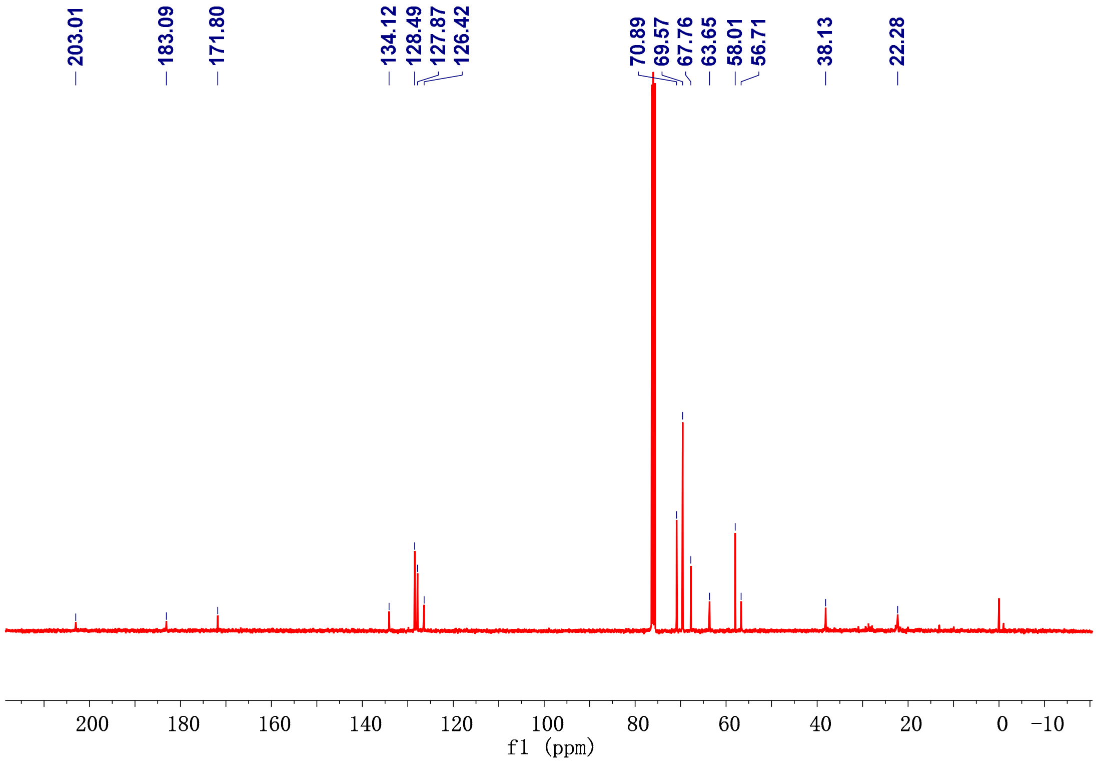

5.2 NMR spectra of Complex 8-15

[$Ru_2(CO)_4(\mu^2$-η^2-$O_2CC_6H_5)_2$ (η^1-$NH_2CH_2C(=O)OCH_2CH_2OCH_3)_2$] (8)

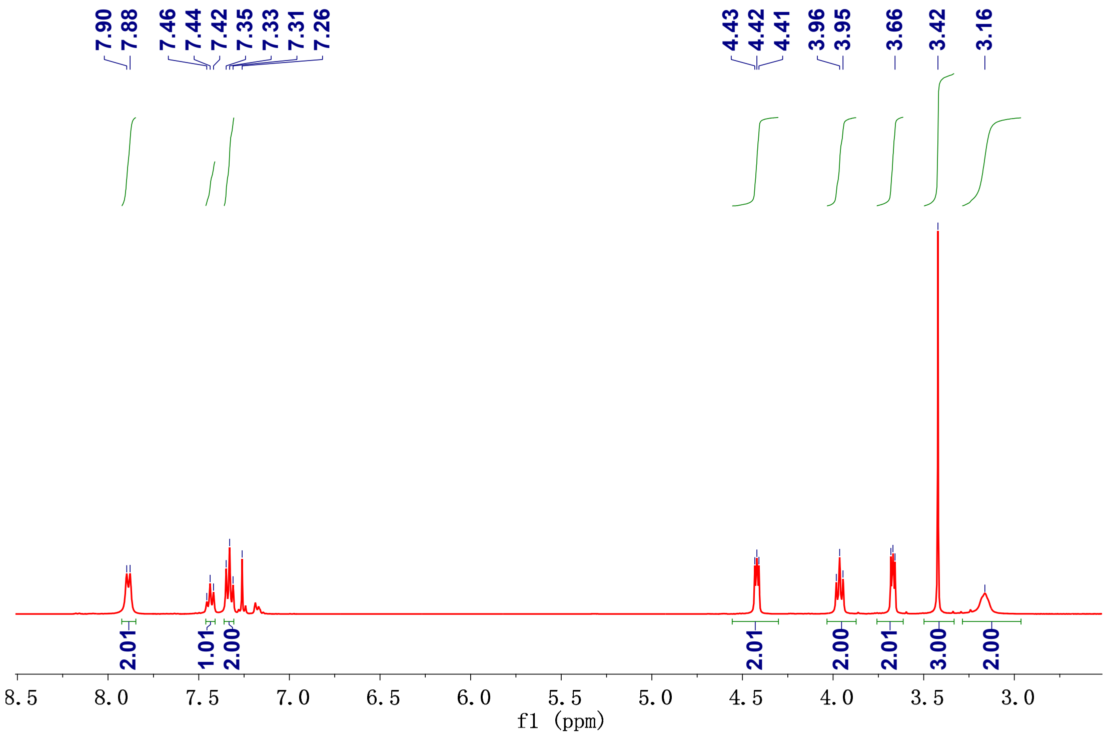

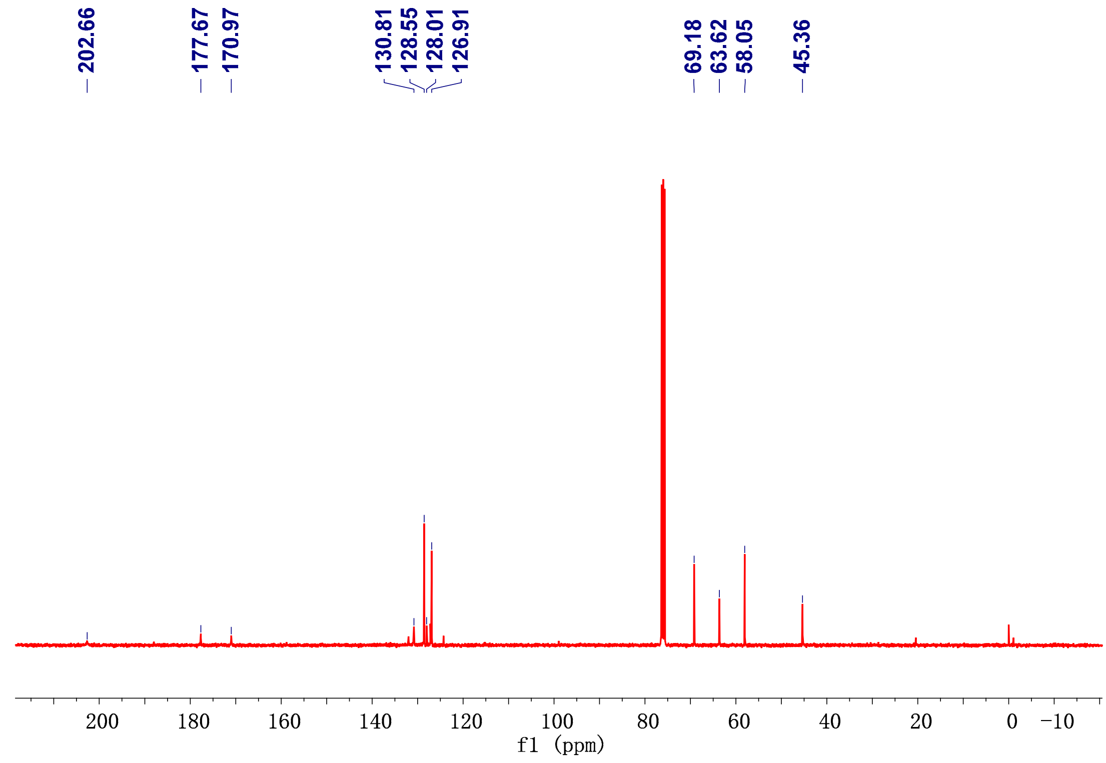

$[Ru_2(CO)_4(\mu^2\text{-}\eta^2\text{-}O_2C\text{-}o\text{-}CH_3C_6H_5)_2(\eta^1\text{-}NH_2CH_2C(=O)OCH_2CH_2OCH_3)_2]$ (9)

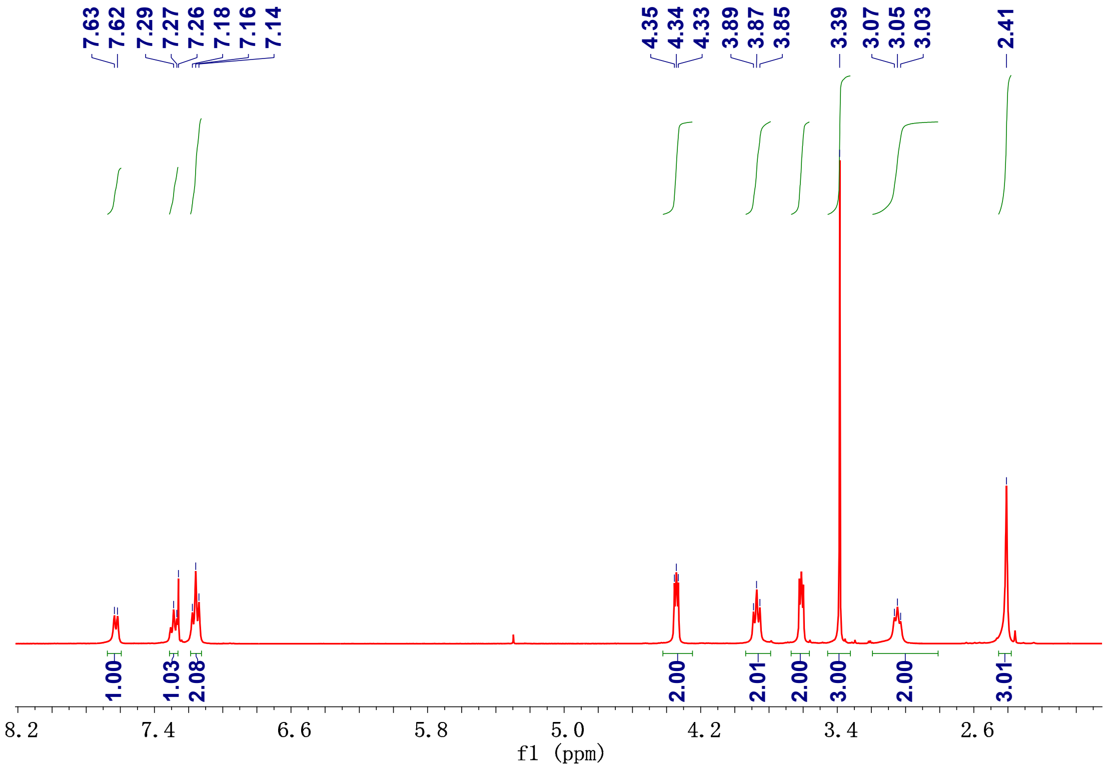

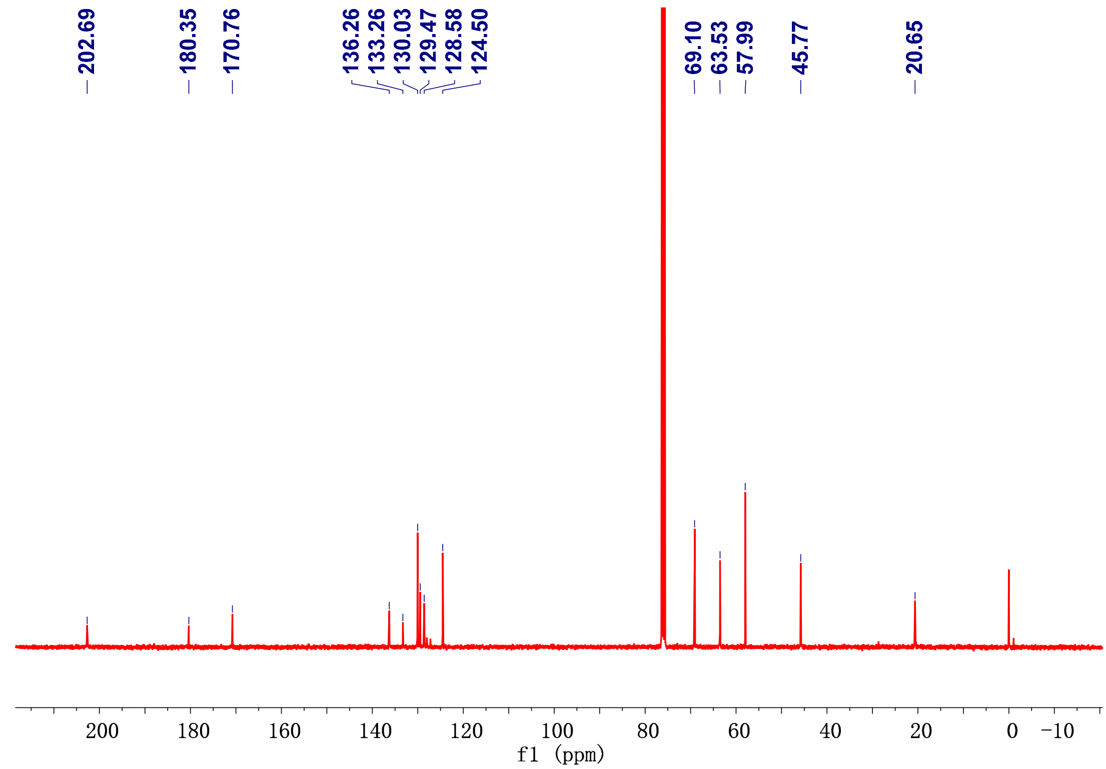

[$Ru_2(CO)_4(\mu^2\text{-}\eta^2\text{-}O_2C\text{-}m\text{-}CH_3C_6H_5)_2$ (η^1-$NH_2CH_2C(=O)OCH_2CH_2OCH_3)_2$] (10)

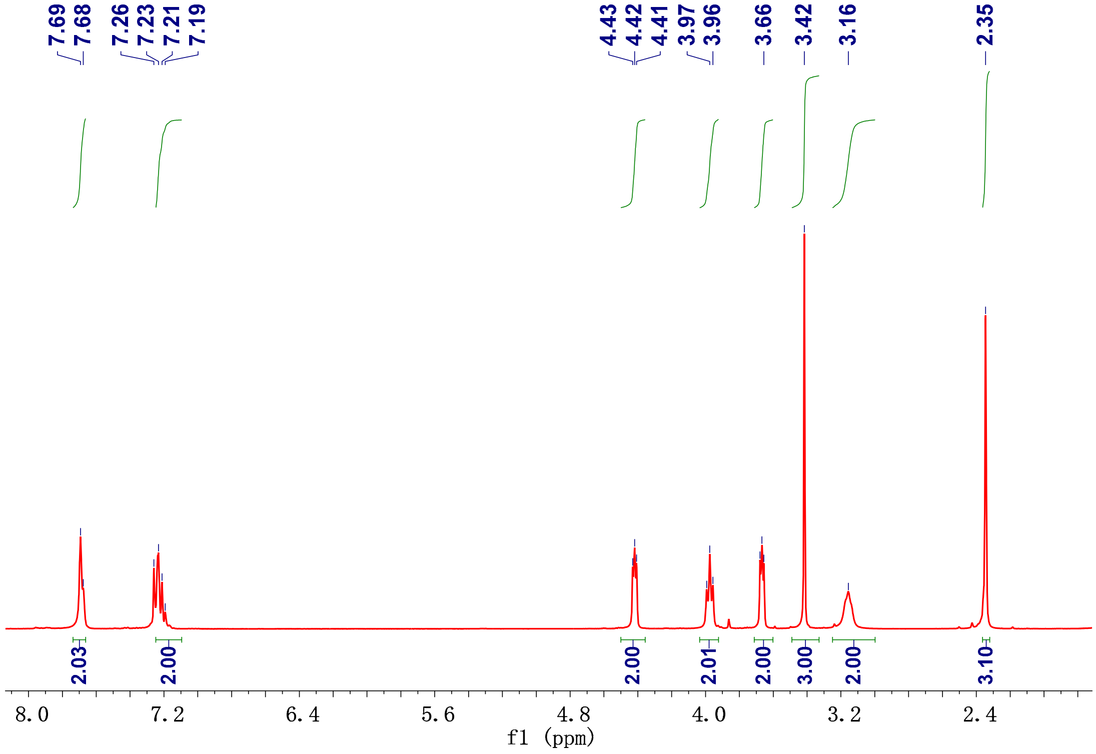

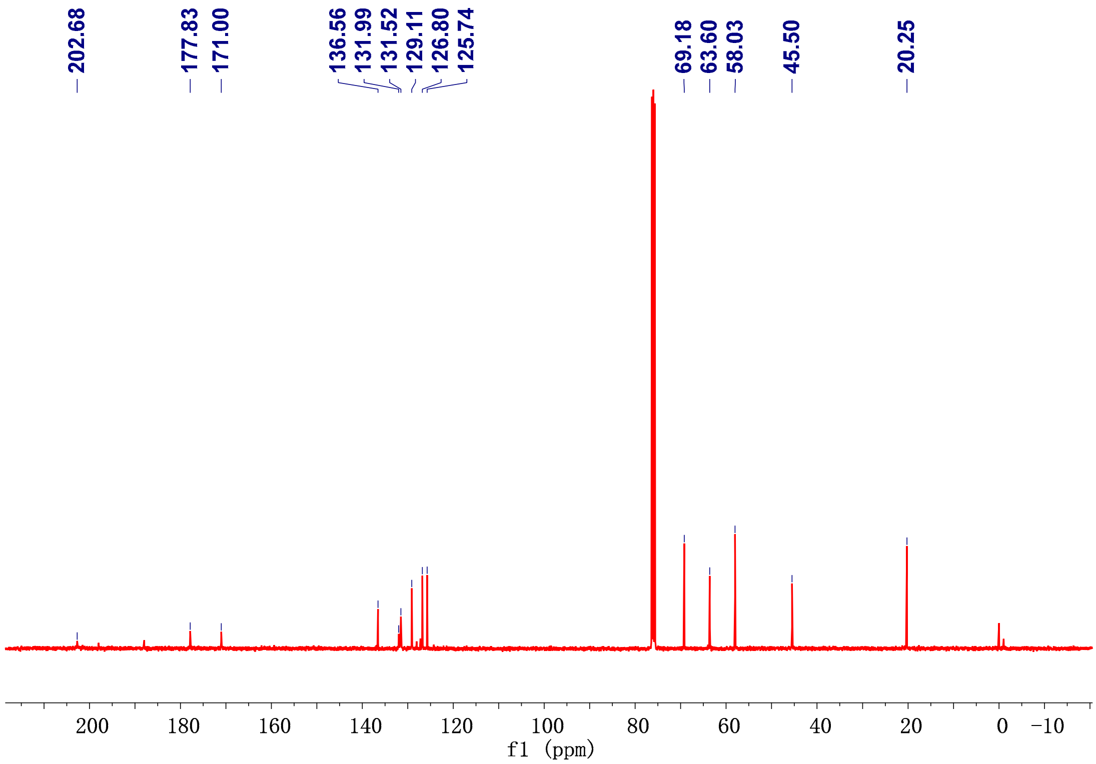

$[Ru_2(CO)_4(\mu^2\text{-}\eta^2\text{-}O_2C\text{-}p\text{-}CH_3C_6H_5)_2\ (\eta^1\text{-}NH_2CH_2C(=O)OCH_2CH_2OCH_3)_2]$ (11)

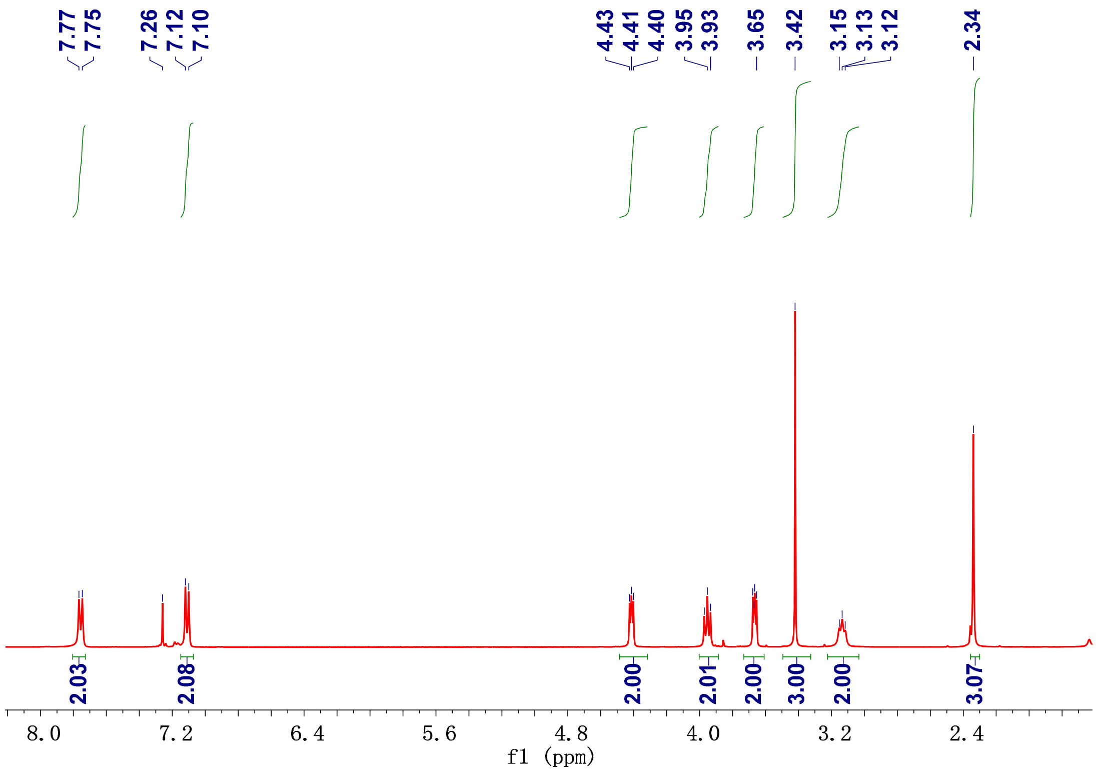

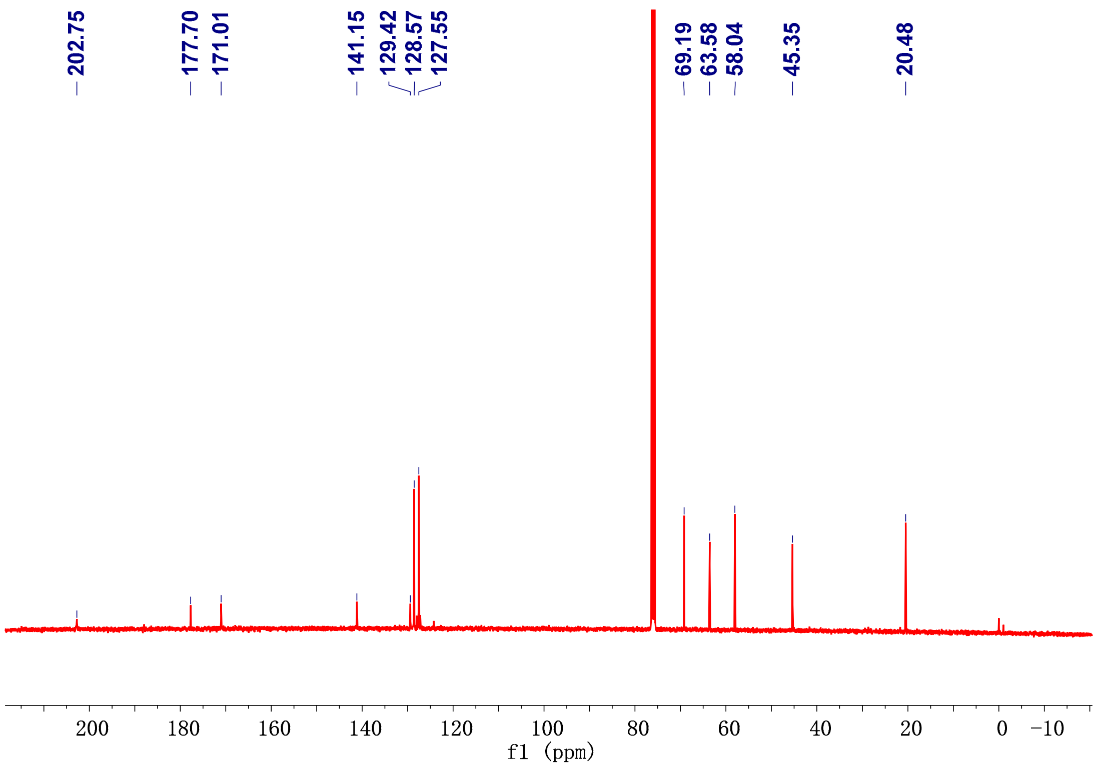

[$Ru_2(CO)_4(\mu^2-\eta^2-O_2C-o-OCH_3C_6H_5)_2(\eta^1-NH_2CH_2C(=O)OCH_2CH_2OCH_3)_2$] (12)

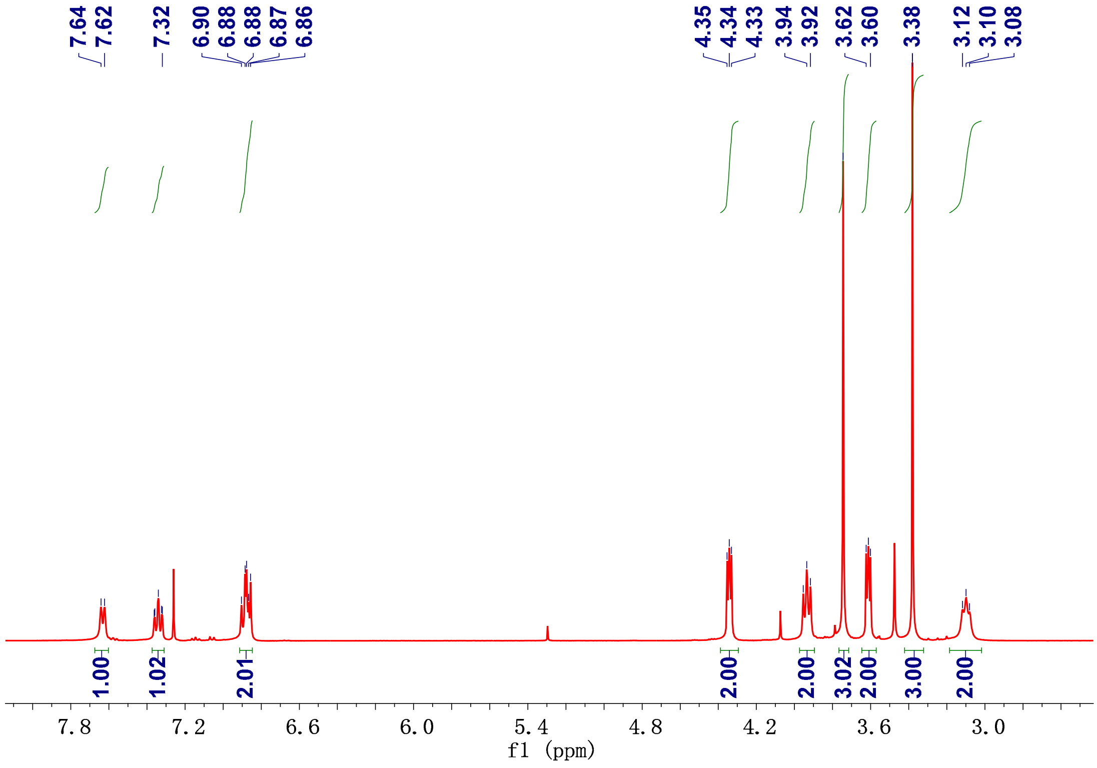

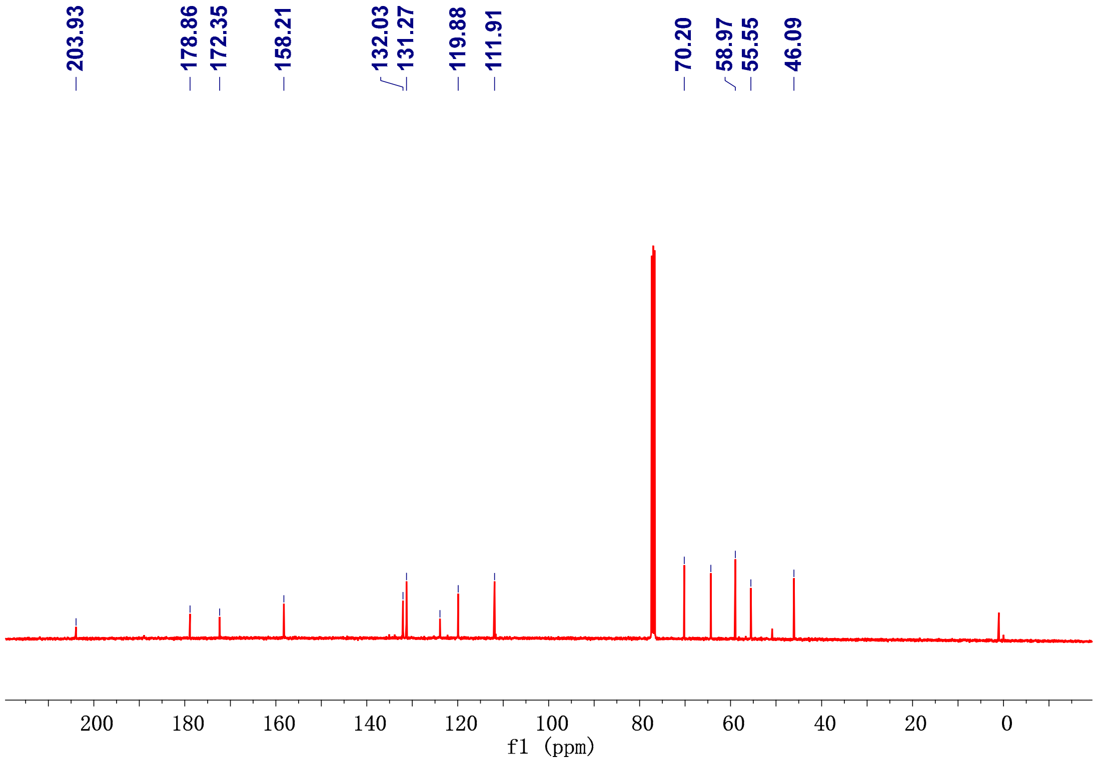

$[Ru_2(CO)_4(\mu^2\text{-}\eta^2\text{-}O_2C\text{-}p\text{-}ClC_6H_5)_2\ (\eta^1\text{-}NH_2CH_2C(=O)OCH_2CH_2OCH_3)_2]$ (13)

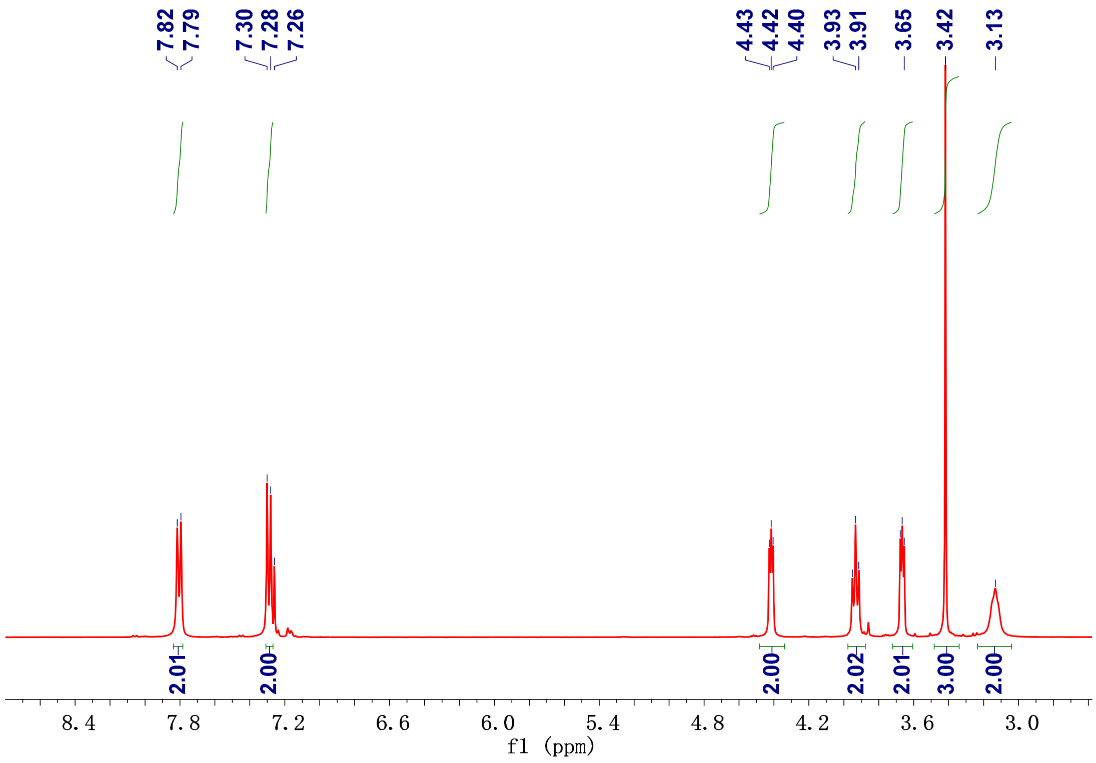

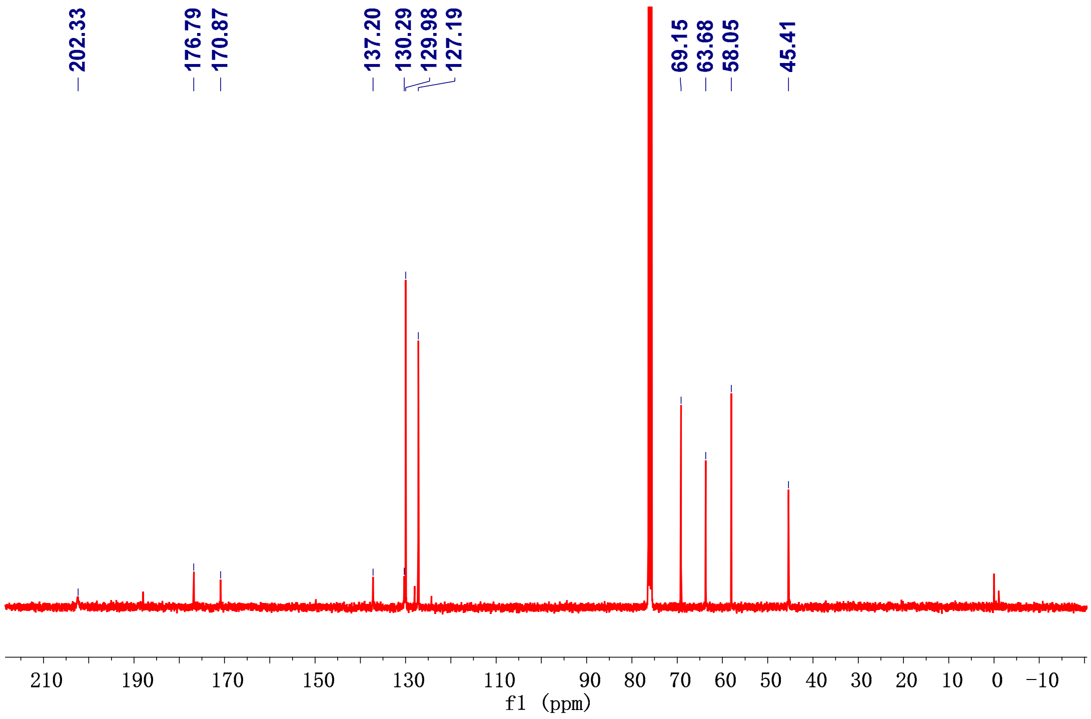

$[Ru_2(CO)_4(\mu^2\text{-}\eta^2\text{-}O_2C\text{-}C_{10}H_7)_2\ (\eta^1\text{-}NH_2CH_2C(=O)OCH_2CH_2OCH_3)_2]$ (14)

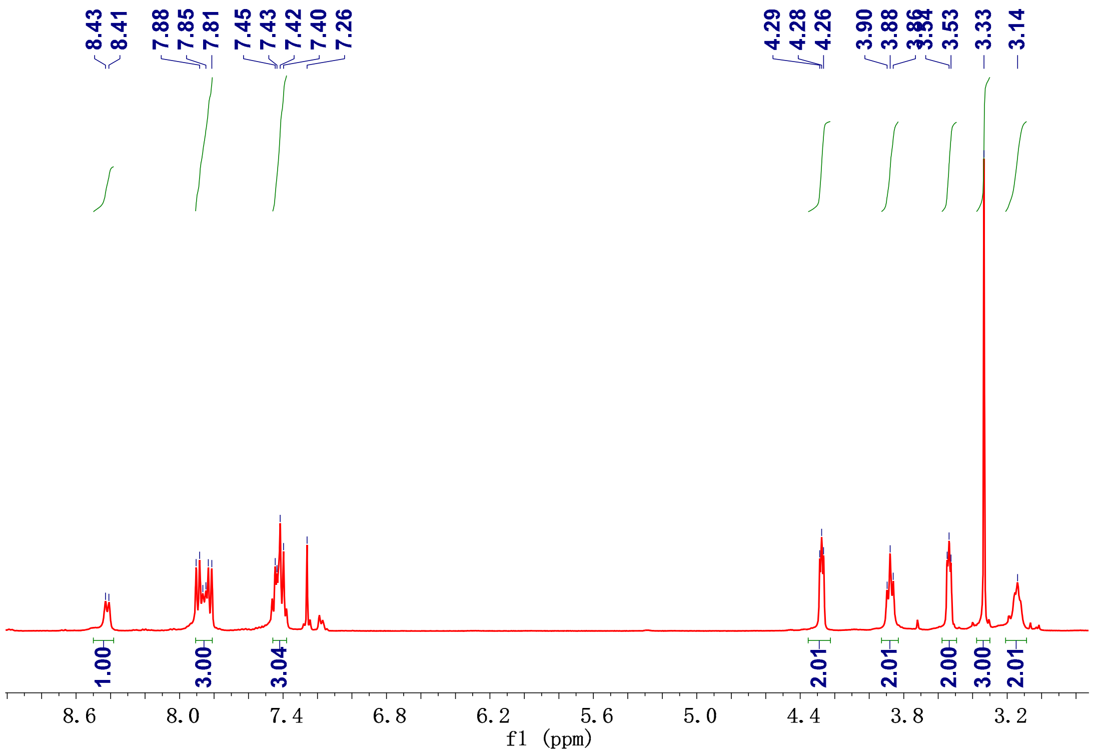

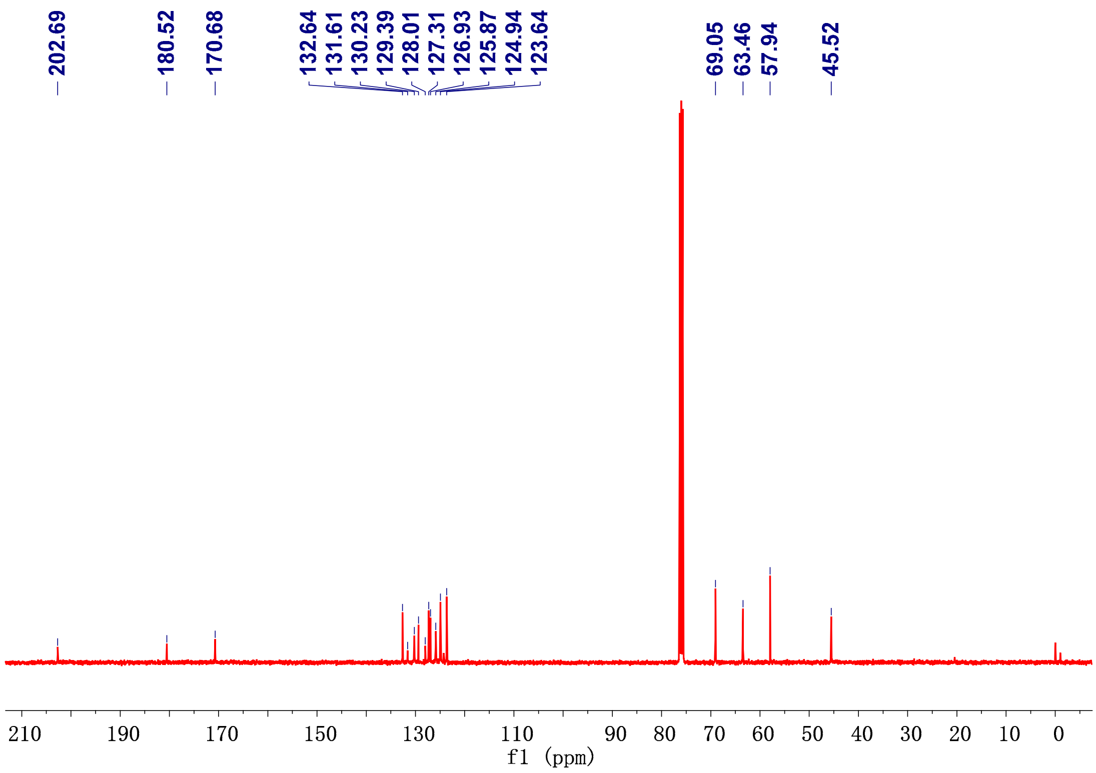

[$Ru_2(CO)_4(\mu^2\text{-}\eta^2\text{-}O_2C\text{-}C_{14}H_9)_2(\eta^1\text{-}NH_2CH_2C(=O)OCH_2CH_2OCH_3)_2$] (15)

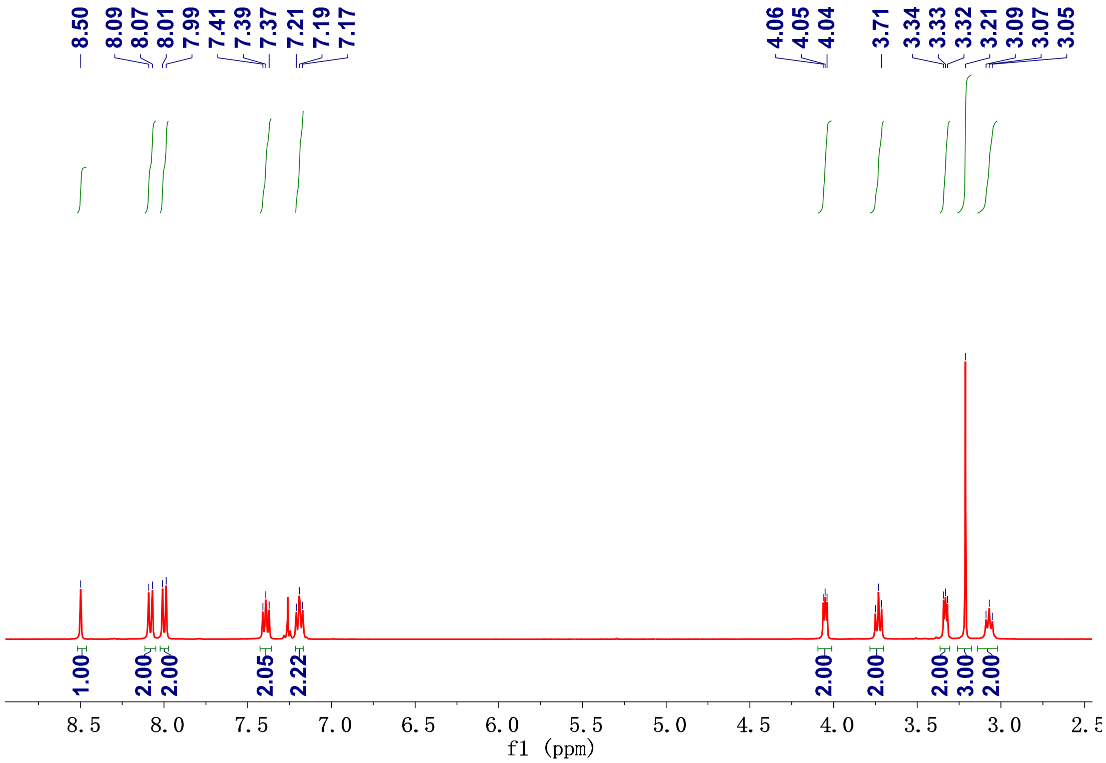

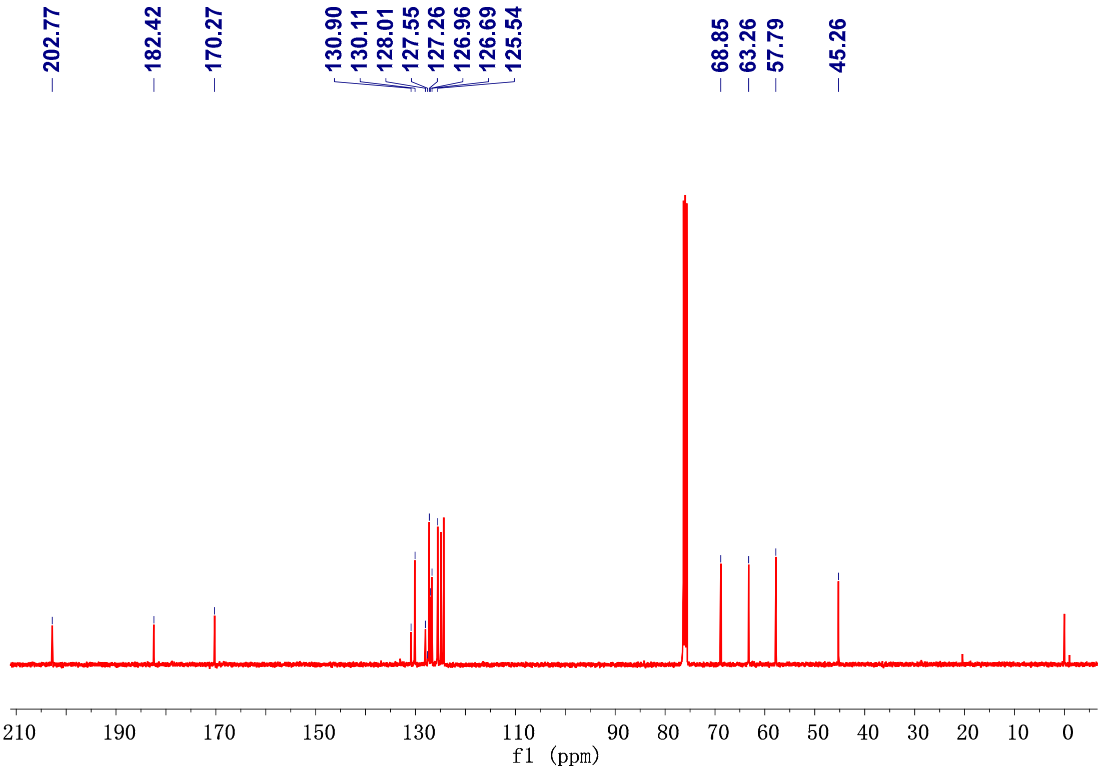

Figure S3: NMR spectra of complex **4**–**15**.

5.3 UV-vis spectra of CO releasing experiments

A typical series of spectra showing the conversion of deoxy-Mb to Mb-CO during light-induced CO dissociation in Figure 4-(a), (b), (c).The UV-vis spectra therefore allow a quantification of the amount of CO released from the CORM with time in Figure 4-(d).

[$Ru_2(CO)_4(\mu^2$-η^2-$O_2CCH_3)_2$ (η^1-$NH_2CH_2C(=O)OCH_2CH_2OCH_3)_2$] (4)

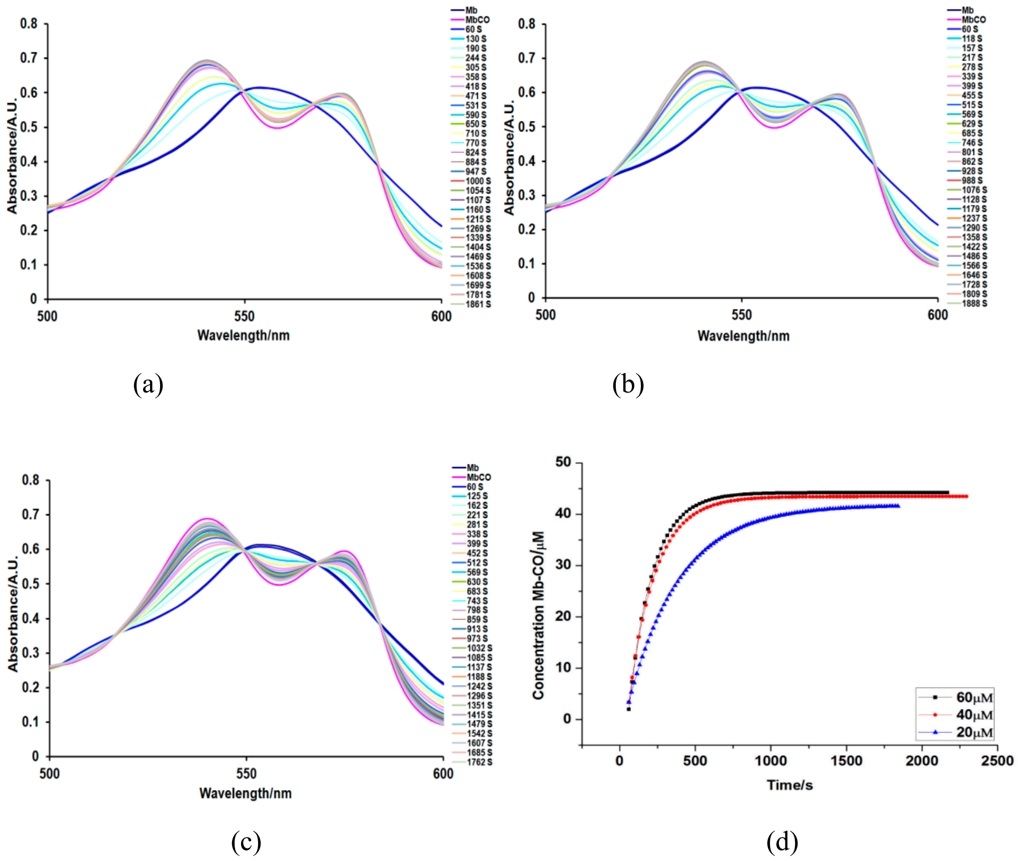

Figure S4-1 Photo-activated CO release profile for **4** (a), (b), (c) UV-vis spectrum showing the Q-bands during the conversion of deoxy-Mb to Mb-CO with time while the concentration of CORMs is 60, 40, 20 μM. (d) The CO-releasing kinetics of **4** in which [Mb-CO] was plotted with CORM at 60, 40, 20μM against time.

$[Ru_2(CO)_4(\mu^2\text{-}\eta^2\text{-}O_2CCH_3)_2\ (\eta^1\text{-}NH_2CH(CH_2Ph)C(=O)OCH_2CH_2OCH_3)_2]$ (5)

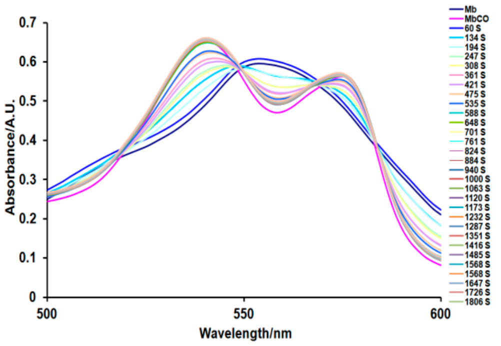

(a)

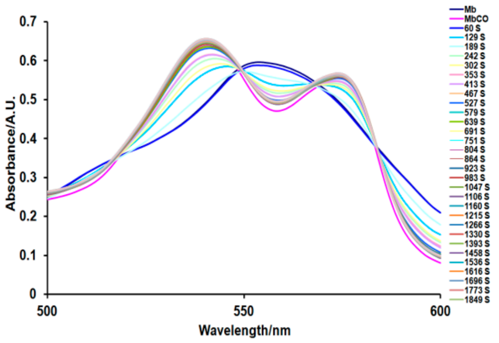

(b)

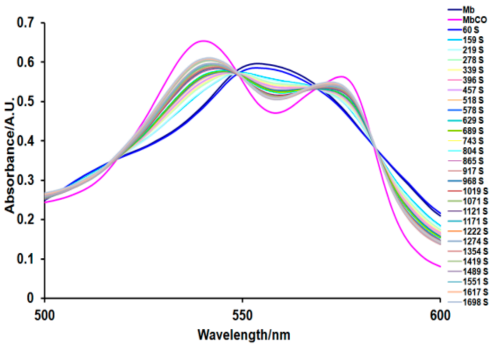

(c)

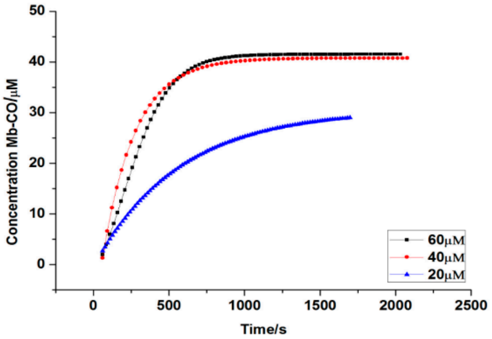

(d)

Figure S4-2 Photo-activated CO release profile for **5** (a), (b), (c) UV-vis spectrum showing the Q-bands during the conversion of deoxy-Mb to Mb-CO with time while the concentration of CORMs is 60, 40, 20 μM. (d) The CO-releasing kinetics of **5** in which [Mb-CO] was plotted with CORM at 60, 40, 20μM against time.

[Ru$_2$(CO)$_4$(μ^2-η^2-O$_2$CCH$_3$)$_2$ (η^1-NH$_2$CH(CH$_2$Ph)C(=O)O(CH$_2$CH$_2$O)$_2$CH$_3$)$_2$] (6)

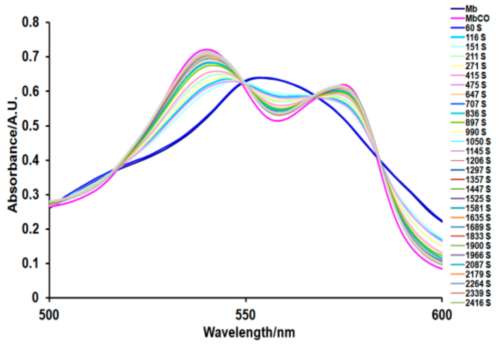

(a)

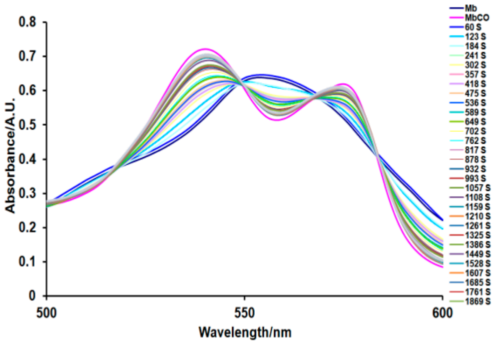

(b)

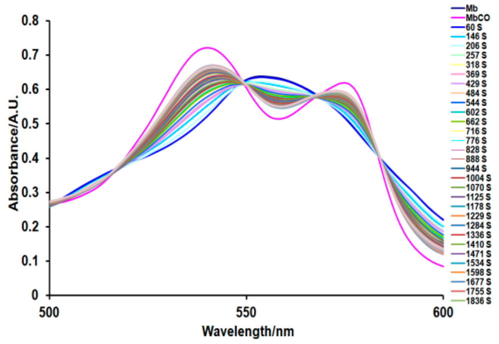

(c)

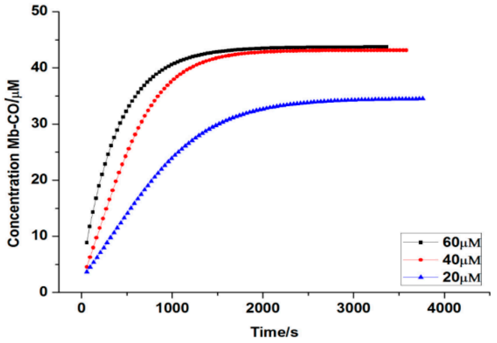

(d)

Figure S4-3 Photo-activated CO release profile for **6** (a), (b), (c) UV-vis spectrum showing the Q-bands during the conversion of deoxy-Mb to Mb-CO with time while the concentration of CORMs is 60, 40, 20 μM. (d) The CO-releasing kinetics of **6** in which [Mb-CO] was plotted with CORM at 60, 40, 20μM against time.

[$Ru_2(CO)_4(\mu^2$-η^2-$O_2CCH_3)_2$ (η^1-$NH_2CH(CH_2Ph)C(=O)O(CH_2CH_2O)_3CH_3)_2$] (7)

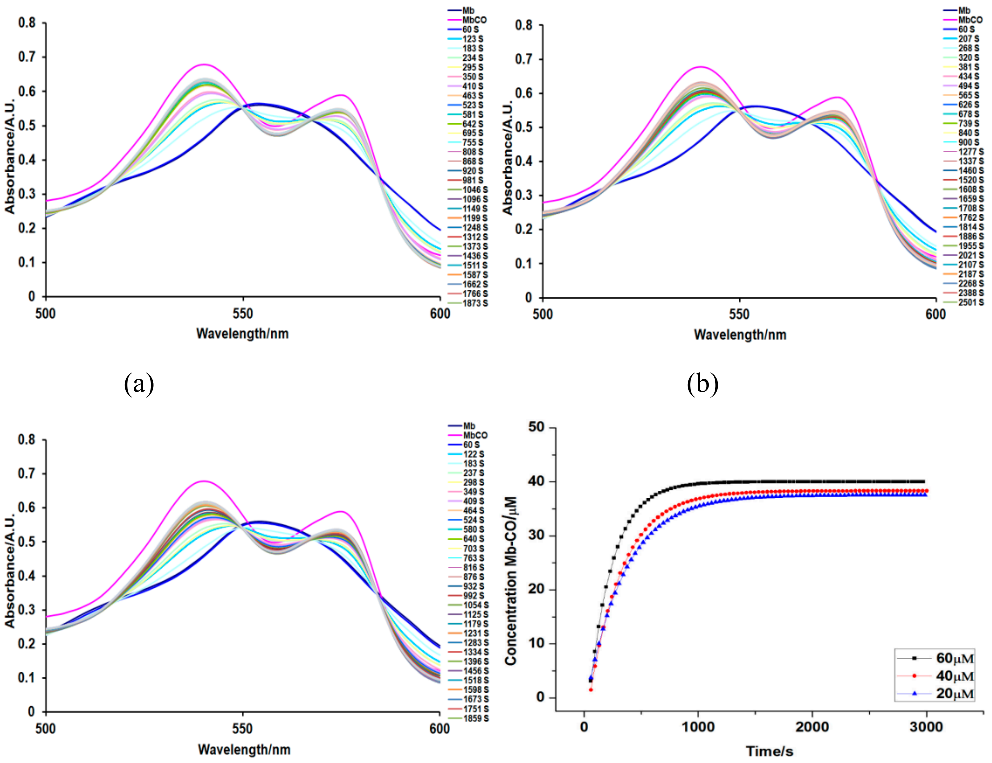

(a) (b)

(c) (d)

Figure S4-4 Photo-activated CO release profile for **7** (a), (b), (c) UV-vis spectrum showing the Q-bands during the conversion of deoxy-Mb to Mb-CO with time while the concentration of CORMs is 60, 40, 20 μM. (d) The CO-releasing kinetics of **7** in which [Mb-CO] was plotted with CORM at 60, 40, 20μM against time.

[$Ru_2(CO)_4(\mu^2\text{-}\eta^2\text{-}O_2CC_6H_5)_2$ (η^1-$NH_2CH_2C(=O)OCH_2CH_2OCH_3)_2$] (8)

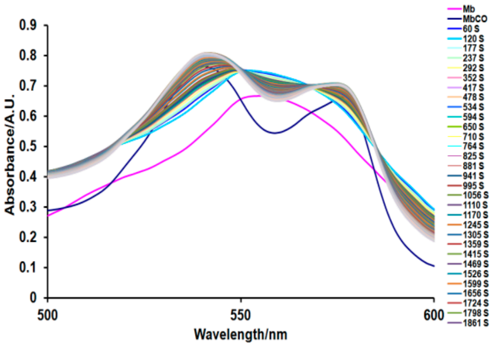

(a)

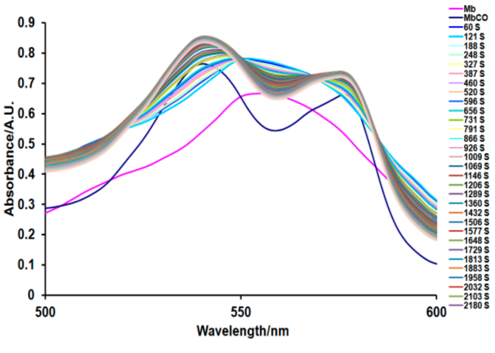

(b)

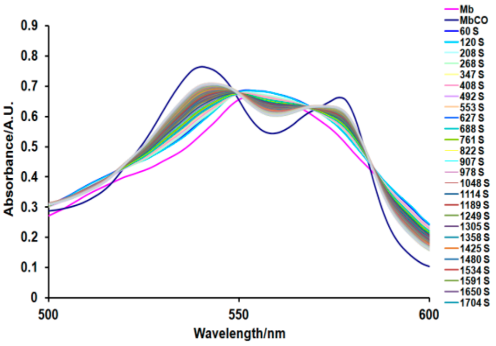

(c)

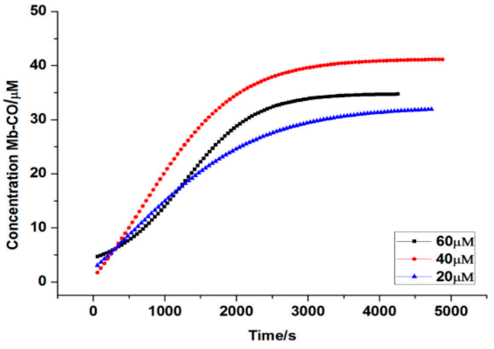

(d)

Figure S4-5 Photo-activated CO release profile for **8** (a), (b), (c) UV-vis spectrum showing the Q-bands during the conversion of deoxy-Mb to Mb-CO with time while the concentration of CORMs is 60, 40, 20 μM. (d) The CO-releasing kinetics of **8** in which [Mb-CO] was plotted with CORM at 60, 40, 20μM against time.

[$Ru_2(CO)_4(\mu^2$-η^2-O_2C-o-$CH_3C_6H_5)_2$ (η^1-$NH_2CH_2C(=O)OCH_2CH_2OCH_3)_2$] (9)

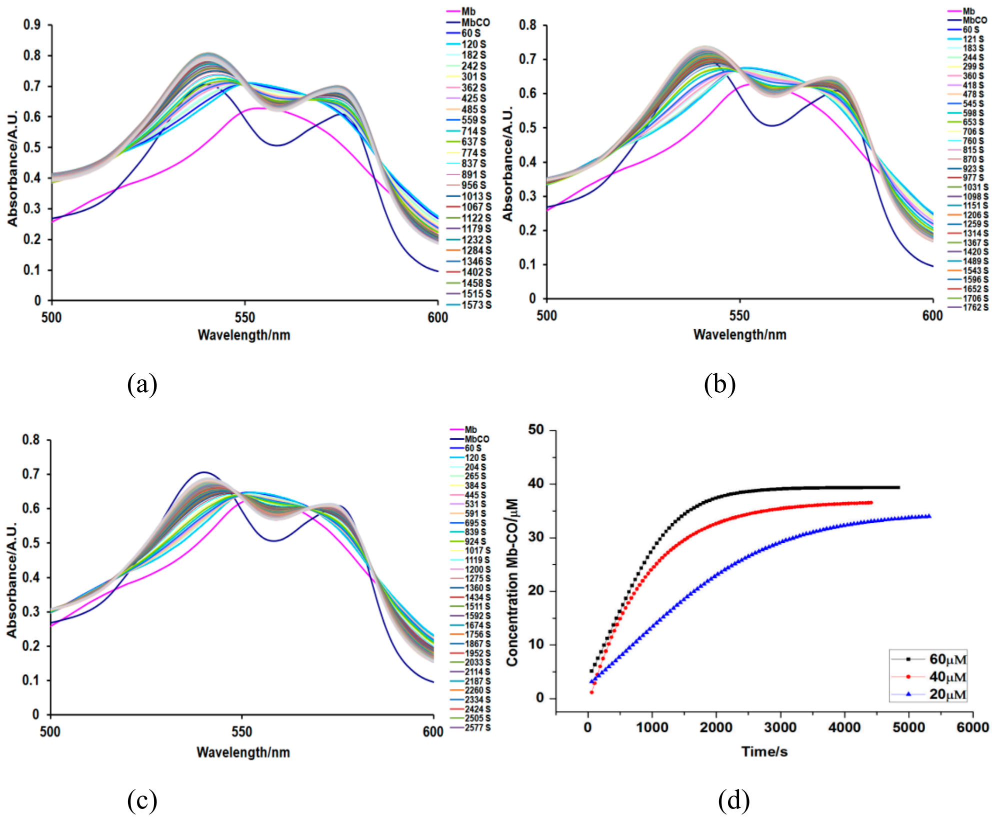

Figure S4-6 Photo-activated CO release profile for **9** (a), (b), (c) UV-vis spectrum showing the Q-bands during the conversion of deoxy-Mb to Mb-CO with time while the concentration of CORMs is 60, 40, 20 µM. (d) The CO-releasing kinetics of **9** in which [Mb-CO] was plotted with CORM at 60, 40, 20µM against time.

$[Ru_2(CO)_4(\mu^2\text{-}\eta^2\text{-}O_2C\text{-}m\text{-}CH_3C_6H_5)_2\ (\eta^1\text{-}NH_2CH_2C(=O)OCH_2CH_2OCH_3)_2]$ (10)

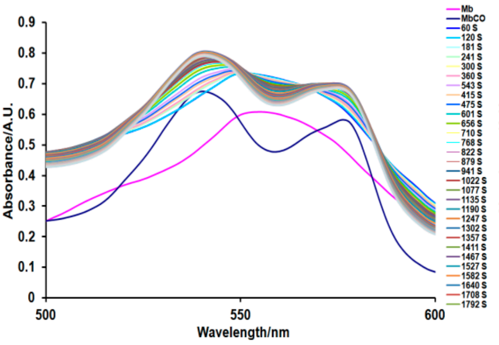

(a)

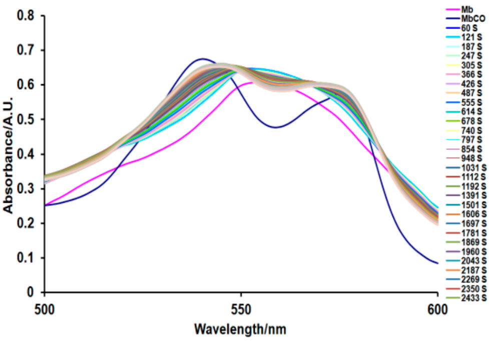

(b)

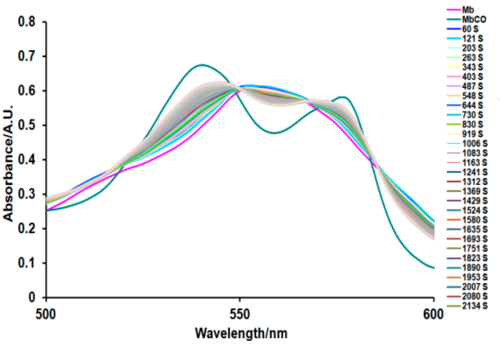

(c)

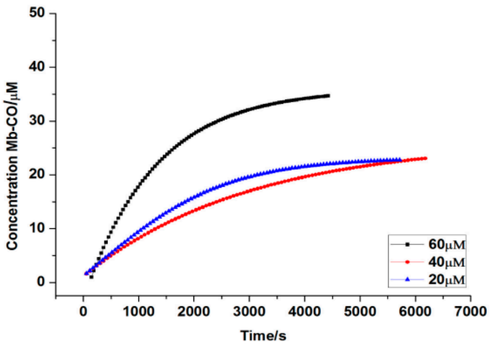

(d)

Figure S4-7 Photo-activated CO release profile for **10** (a), (b), (c) UV-vis spectrum showing the Q-bands during the conversion of deoxy-Mb to Mb-CO with time while the concentration of CORMs is 60, 40, 20 μM. (d) The CO-releasing kinetics of **10** in which [Mb-CO] was plotted with CORM at 60, 40, 20μM against time.

$[Ru_2(CO)_4(\mu^2\text{-}\eta^2\text{-}O_2C\text{-}p\text{-}CH_3C_6H_5)_2\ (\eta^1\text{-}NH_2CH_2C(=O)OCH_2CH_2OCH_3)_2]$ (11)

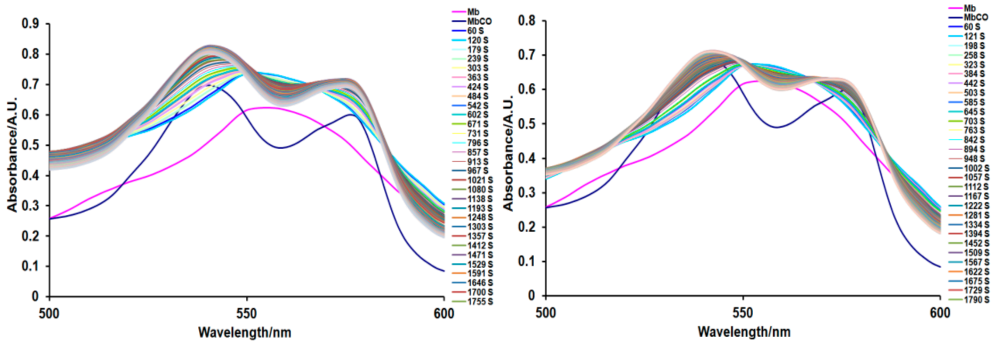

(a) (b)

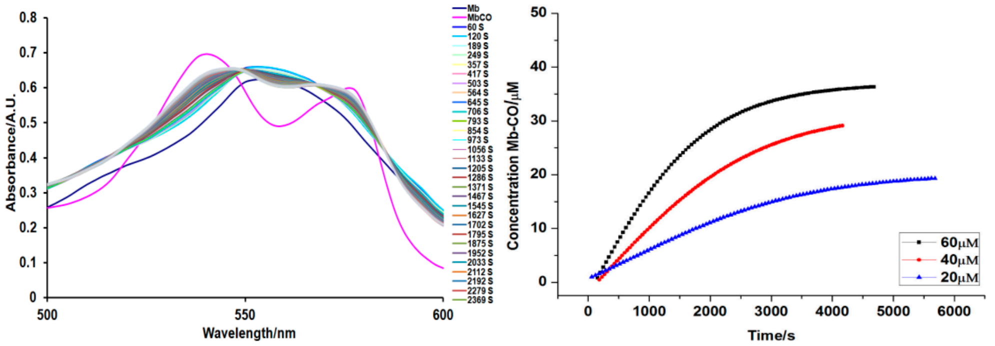

(c) (d)

Figure S4-8 Photo-activated CO release profile for **11** (a), (b), (c) UV-vis spectrum showing the Q-bands during the conversion of deoxy-Mb to Mb-CO with time while the concentration of CORMs is 60, 40, 20 μM. (d) The CO-releasing kinetics of **11** in which [Mb-CO] was plotted with CORM at 60, 40, 20μM against time.

$[Ru_2(CO)_4(\mu^2\text{-}\eta^2\text{-}O_2C\text{-}o\text{-}OCH_3C_6H_5)_2(\eta^1\text{-}NH_2CH_2C(=O)OCH_2CH_2OCH_3)_2]$ (12)

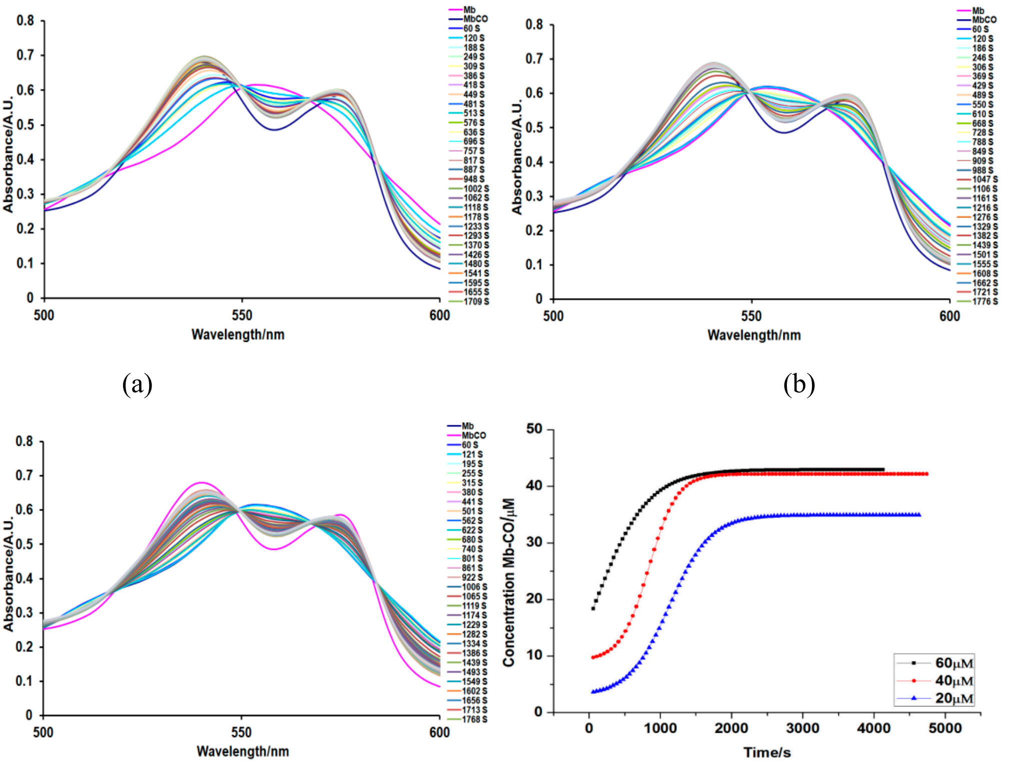

(a) (b)

(c) (d)

Figure S4-9 Photo-activated CO release profile for **12** (a), (b), (c) UV-vis spectrum showing the Q-bands during the conversion of deoxy-Mb to Mb-CO with time while the concentration of CORMs is 60, 40, 20 μM. (d) The CO-releasing kinetics of **12** in which [Mb-CO] was plotted with CORM at 60, 40, 20μM against time.

$[Ru_2(CO)_4(\mu^2\text{-}\eta^2\text{-}O_2C\text{-}p\text{-}ClC_6H_5)_2\ (\eta^1\text{-}NH_2CH_2C(=O)OCH_2CH_2OCH_3)_2]$ (13)

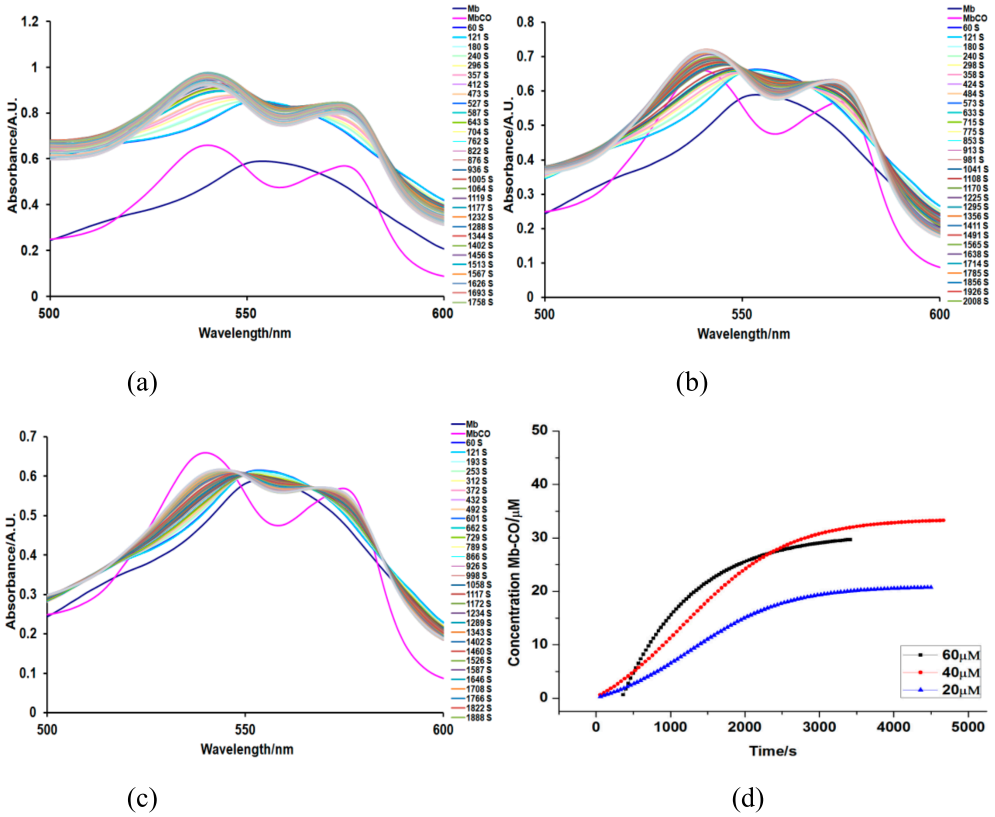

Figure S4-10 Photo-activated CO release profile for **13** (a), (b), (c) UV-vis spectrum showing the Q-bands during the conversion of deoxy-Mb to Mb-CO with time while the concentration of CORMs is 60, 40, 20 μM. (d) The CO-releasing kinetics of **13** in which [Mb-CO] was plotted with CORM at 60, 40, 20μM against time.

[$Ru_2(CO)_4(\mu^2$-η^2-O_2C-$C_{10}H_7)_2$ (η^1-$NH_2CH_2C(=O)OCH_2CH_2OCH_3)_2$] (14)

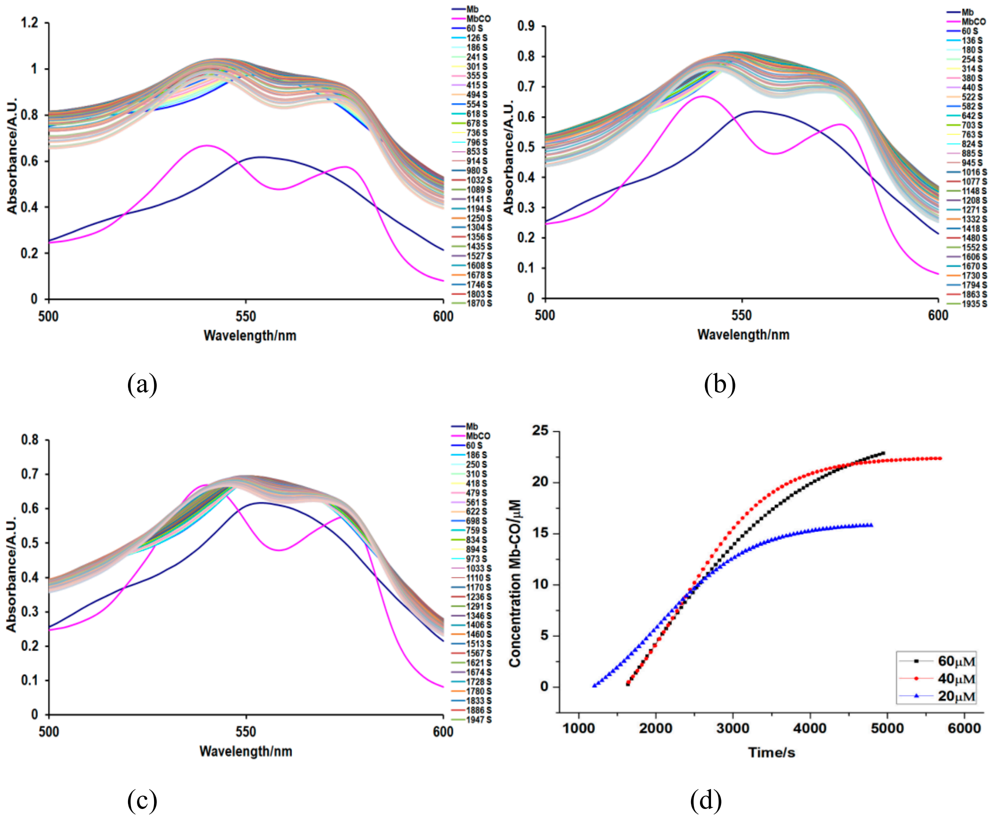

Figure S4-11 Photo-activated CO release profile for 1**4** (a), (b), (c) UV-vis spectrum showing the Q-bands during the conversion of deoxy-Mb to Mb-CO with time while the concentration of CORMs is 60, 40, 20 μM. (d) The CO-releasing kinetics of 1**4** in which [Mb-CO] was plotted with CORM at 60, 40, 20μM against time.

[$Ru_2(CO)_4(\mu^2$-η^2-O_2C-$C_{14}H_9)_2$ (η^1-$NH_2CH_2C(=O)OCH_2CH_2OCH_3)_2$] (15)

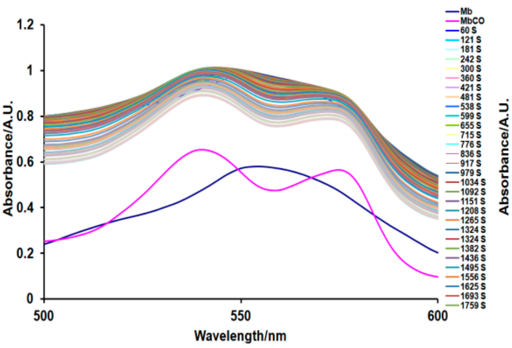

(a)

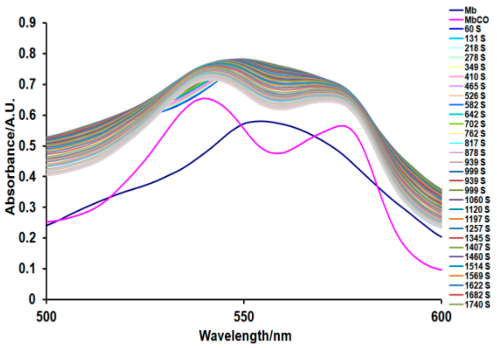

(b)

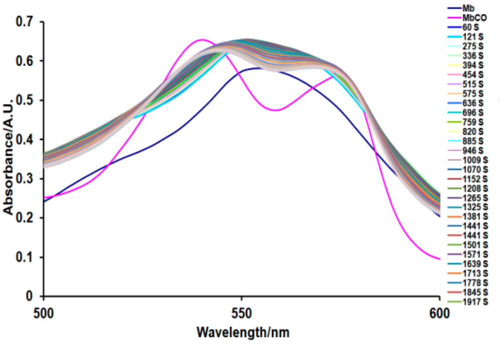

(c)

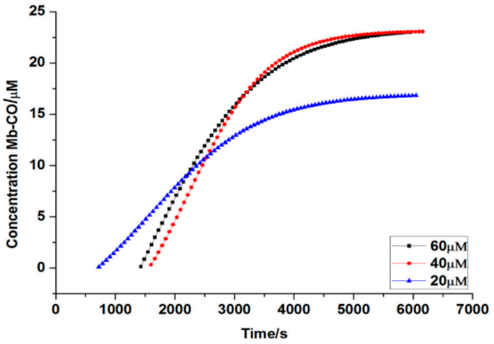

(d)

Figure S4-12 Photo-activated CO release profile for **15** (a), (b), (c) UV-vis spectrum showing the Q-bands during the conversion of deoxy-Mb to Mb-CO with time while the concentration of CORMs is 60, 40, 20 μM. (d) The CO-releasing kinetics of **15** in which [Mb-CO] was plotted with CORM at 60, 40, 20μM against time.

5.4 UV-vis spectra of Lipophilicity (log $P_{o/w}$)

Table S3. Stand curve measurement and calculation of Log P

Entry	Standard curve equation	Correlation coefficient（R^2）	C (mmol/L)		logP
			$C_{octanol}$	C_{water}	
4	A=11.662C+0.267	0.99211	0.5665	0.2315	0.39
5	A=18.489C-0.386	0.99857	0.9852	0.03845	1.41
6	A=11.484C+0.100	0.99574	0.8156	0.0549	1.17
7	A=14.278C+0.217	0.98619	0.6997	0.06228	1.05
8	A=10.859C+0.123	0.98815	1.1277	0.0222	1.71
9	A=10.433C+0.371	0.988	1.0835	0.02325	1.67
10	A=12.439C+0.239	0.99086	0.8547	0.08045	1.03
11	A=11.640C+0.113	0.99014	0.8735	0.07575	1.06
12	A=4.096C-0.0221	0.99651	1.0636	0.01755	1.78
13	A=11.366C+0.260	0.99156	0.9201	0.05055	1.26

$[Ru_2(CO)_4(\mu^2\text{-}\eta^2\text{-}O_2CCH_3)_2(\eta^1\text{-}NH_2CH_2C(=O)OCH_2CH_2OCH_3)_2]$ (4)

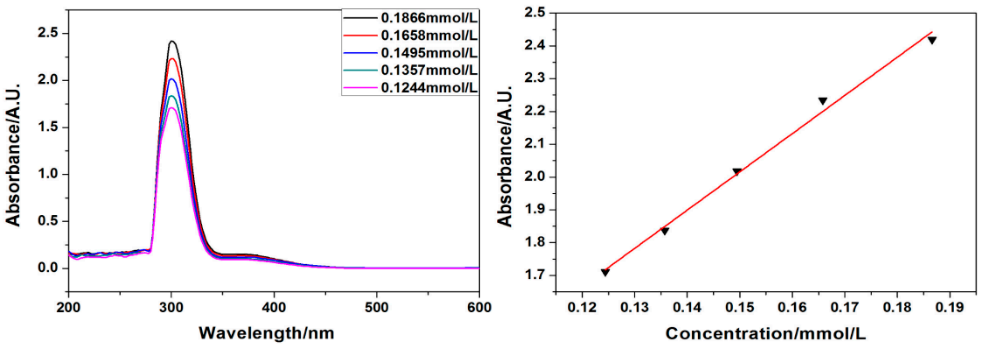

Figure S5 UV-vis spectrum showing the Q-bands during different concentrations of **4** (left) and Standard curve of **4** (right).

$[Ru_2(CO)_4(\mu^2\text{-}\eta^2\text{-}O_2CCH_3)_2(\eta^1\text{-}NH_2CH(CH_2Ph)C(=O)OCH_2CH_2OCH_3)_2]$ (5)

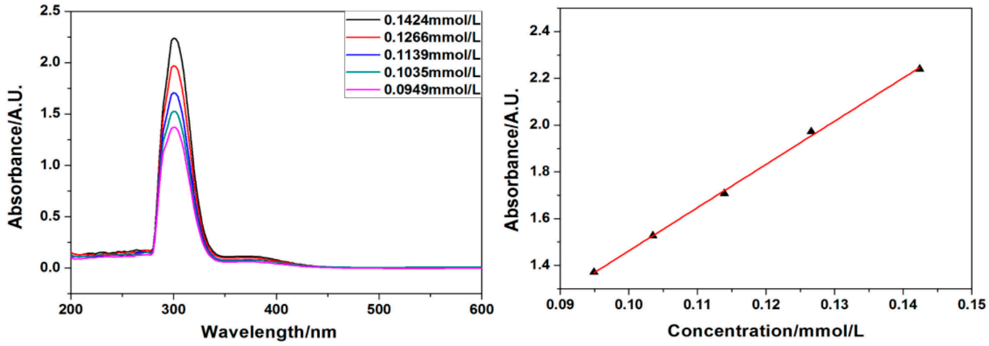

Figure S6 UV-vis spectrum showing the Q-bands during different concentrations of **5** (left) and Standard curve of **5** (right).

$[Ru_2(CO)_4(\mu^2\text{-}\eta^2\text{-}O_2CCH_3)_2(\eta^1\text{-}NH_2CH(CH_2Ph)C(=O)O(CH_2CH_2O)_2CH_3)_2]$ (6)

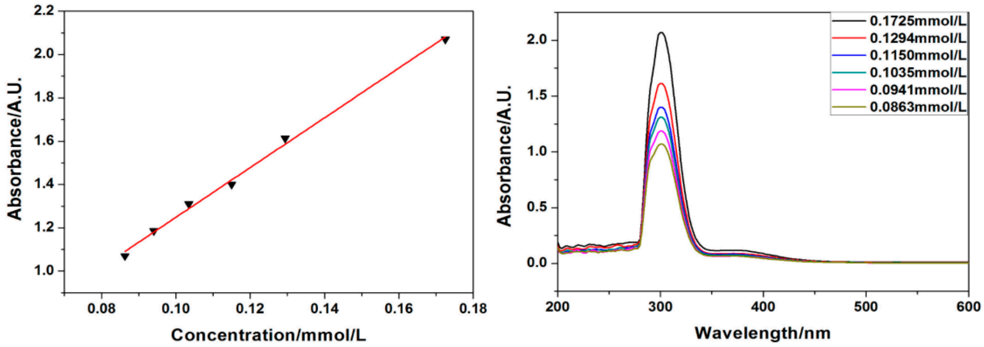

Figure S7 UV-vis spectrum showing the Q-bands during different concentrations of **6** (left) and Standard curve of **6** (right).

[$Ru_2(CO)_4(\mu^2$-η^2-$O_2CCH_3)_2$ (η^1-$NH_2CH(CH_2Ph)C(=O)O(CH_2CH_2O)_3CH_3)_2$] (7)

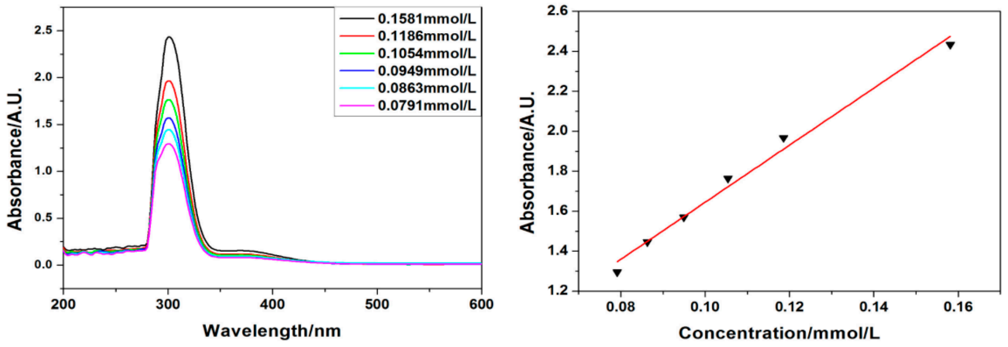

Figure S8 UV-vis spectrum showing the Q-bands during different concentrations of **7** (left) and Standard curve of **7** (right).

[$Ru_2(CO)_4(\mu^2$-η^2-$O_2CC_6H_5)_2$ (η^1-$NH_2CH_2C(=O)OCH_2CH_2OCH_3)_2$] (8)

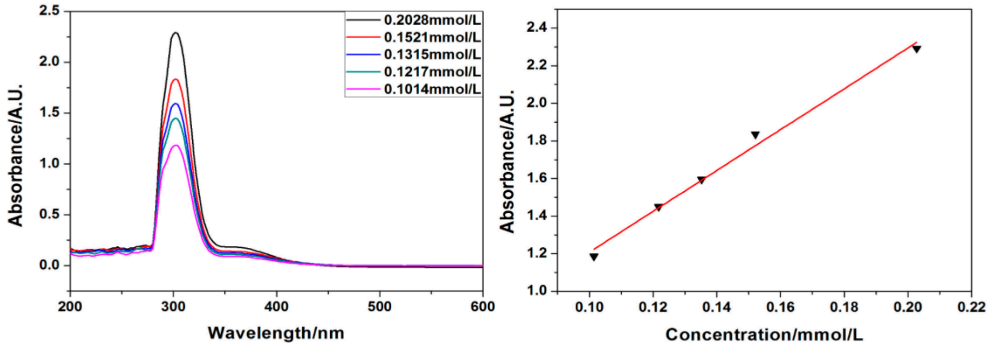

Figure S9 UV-vis spectrum showing the Q-bands during different concentrations of **8** (left) and Standard curve of **8** (right).

[$Ru_2(CO)_4(\mu^2$-η^2-O_2C-o-$CH_3C_6H_5)_2$ (η^1-$NH_2CH_2C(=O)OCH_2CH_2OCH_3)_2$] (9)

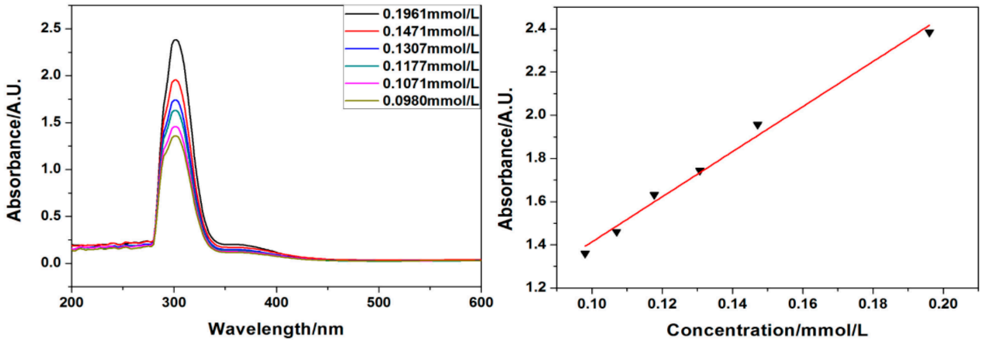

Figure S10 UV-vis spectrum showing the Q-bands during different concentrations of **9** (left) and Standard curve of **9** (right).

$[Ru_2(CO)_4(\mu^2\text{-}\eta^2\text{-}O_2C\text{-}m\text{-}CH_3C_6H_5)_2\ (\eta^1\text{-}NH_2CH_2C(=O)OCH_2CH_2OCH_3)_2]$ (10)

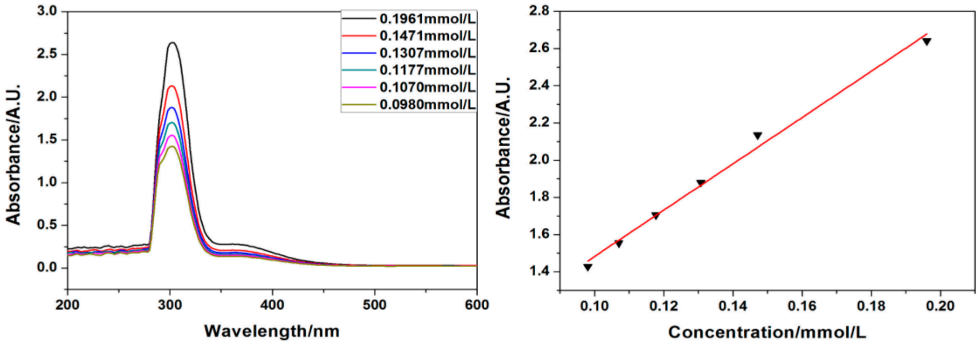

Figure S11 UV-vis spectrum showing the Q-bands during different concentrations of **10** (left) and Standard curve of **10** (right).

$[Ru_2(CO)_4(\mu^2\text{-}\eta^2\text{-}O_2C\text{-}p\text{-}CH_3C_6H_5)_2\ (\eta^1\text{-}NH_2CH_2C(=O)OCH_2CH_2OCH_3)_2]$ (11)

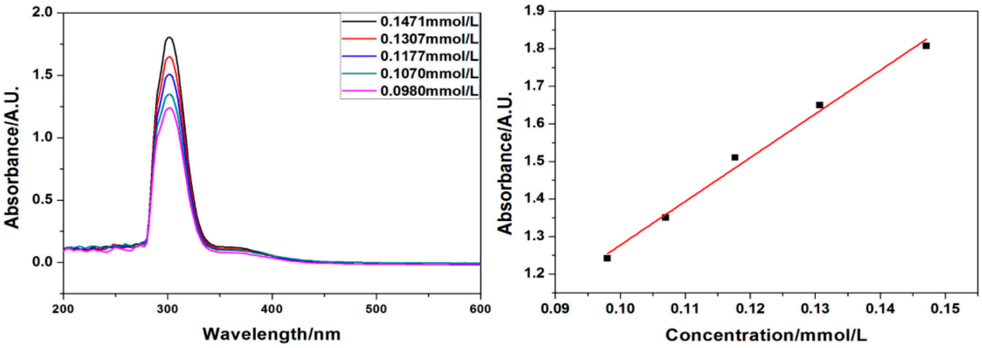

Figure S12 UV-vis spectrum showing the Q-bands during different concentrations of **11** (left) and Standard curve of **11** (right).

$[Ru_2(CO)_4(\mu^2\text{-}\eta^2\text{-}O_2C\text{-}o\text{-}OCH_3C_6H_5)_2\ (\eta^1\text{-}NH_2CH_2C(=O)OCH_2CH_2OCH_3)_2]$ (12)

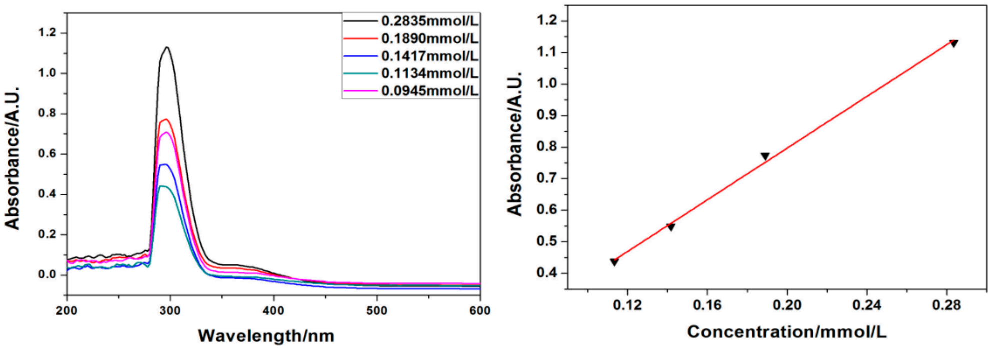

Figure S13 UV-vis spectrum showing the Q-bands during different concentrations of **12** (left) and Standard curve of **12** (right).

$[Ru_2(CO)_4(\mu^2\text{-}\eta^2\text{-}O_2C\text{-}p\text{-}ClC_6H_5)_2\ (\eta^1\text{-}NH_2CH_2C(=O)OCH_2CH_2OCH_3)_2]$ (13)

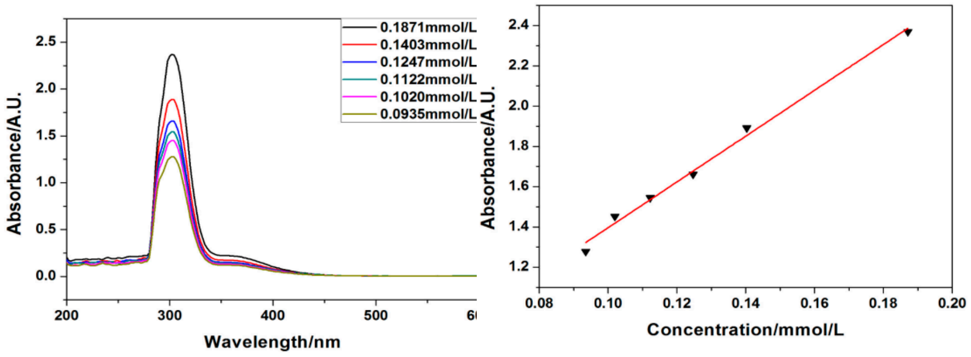

Figure S14 UV-vis spectrum showing the Q-bands during different concentrations of **13** (left) and Standard curve of **13** (right).

6. Cell culture and Cytotoxicity studies

Murine RAW264.7 and HT29 cells were used for the preliminary cytotoxicity studies. The cryopreserved RAW264.7 and HT29 cell lines were quickly thawed at 37 °C, and then centrifugal 5 min at 1000 r/min. The cell lines were cleaned for 1-2 times by FBS medium. The process of frozen joined the harmful substances for the growth of cell reproduction. The sample was centrifuged after resuspensed with FBS medium. The culture medium were added in RAW264.7 cells which changed every day. After cells growth long full which can be used backup.

The stock solutions of CORM **4** and **8** were prepared using DMSO (10 mmol/L and 100 mmol/L). The solution were diluted with DMEM culture medium in the concentration range from 50 μM to 700 μM and 10μM to 50μM. RAW264.7 and HT29 cells were seeded in 96-well plates at 5×10^3 cells per well and the cells were incubated in 5% CO_2 for 24 h at 37 °C. Then, **4** and **8** at different concentrations (150μL) were incubated with RAW264.7 and HT29 cells for 2h in three 96-well plates, respectively. The cells which irradiated for 15min with 365 nm light or not placed in the dark for 8h measured by MTT assay.

Every 96-well plate was added 15 μL MTT, then the cells were incubated in 5% CO_2 for 3-4 h at 37 °C. After Removing the plates liquid, 200μL DMSO was added and the sample tube was shaked for 10 min at room temperature. The OD value of same time was measured using a Enzyme mark detector (Thermo MK3) with an excitation wavelength of 420 nm.

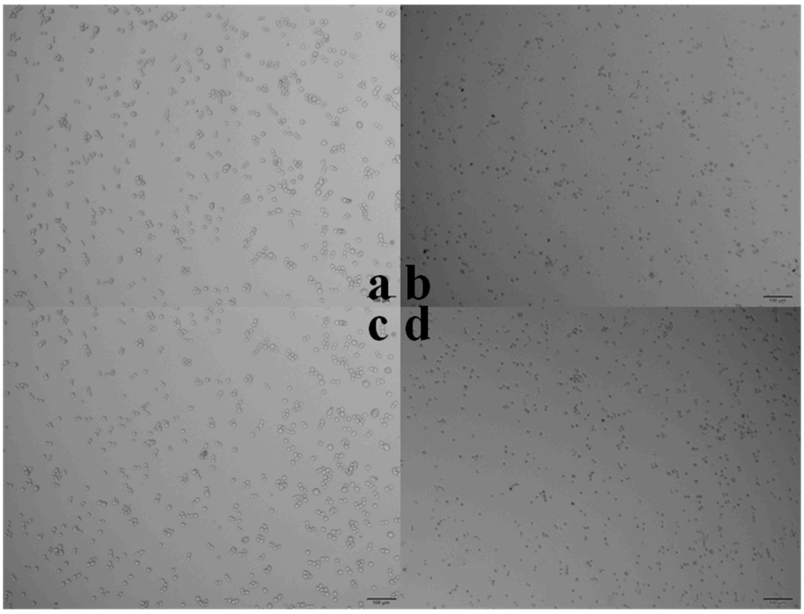

Figure S15 RAW264.7 cell changes inhibited by **4** (dark: a-50 μM； b-500 μM, illumination: c -50 μM; d-500 μM)

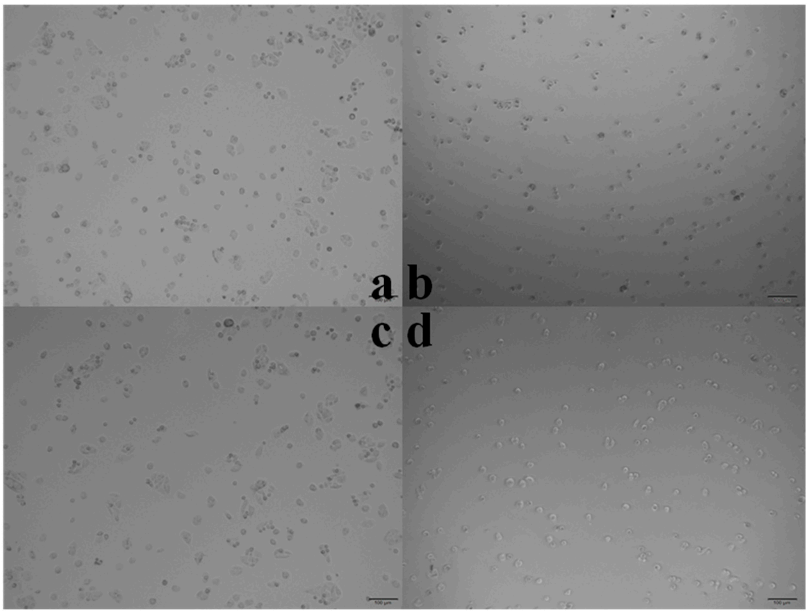

Figure S16 HT29 cell changes inhibited by **4** (dark: a-50 μM； b-500 μM, illumination: c -50 μM; d-500 μM)

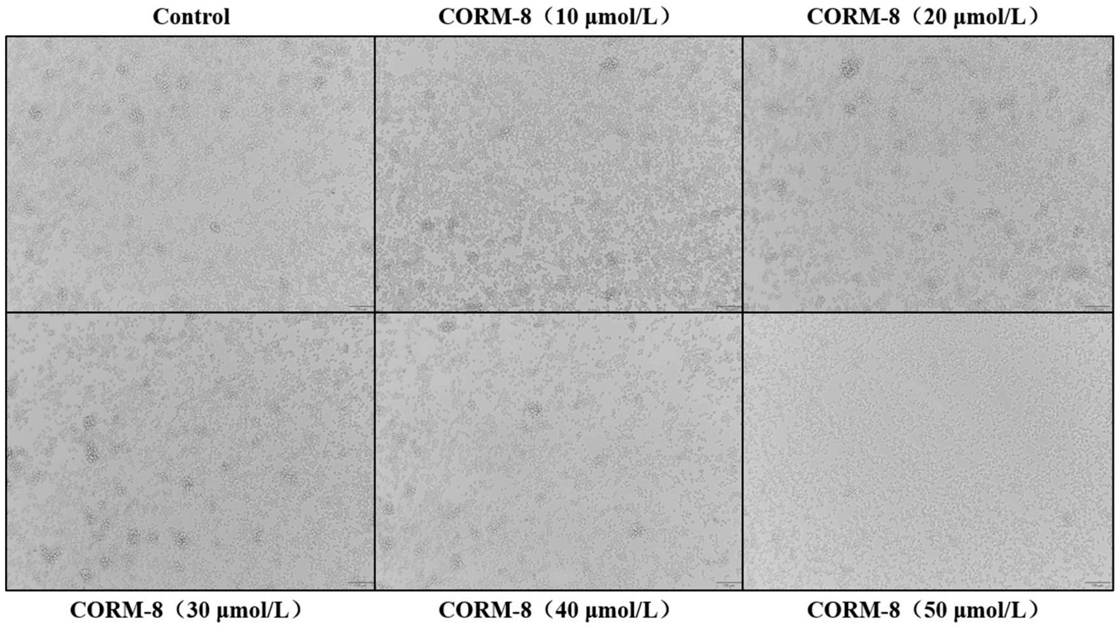

Figure S17 RAW264.7 cell changes inhibited by **8**

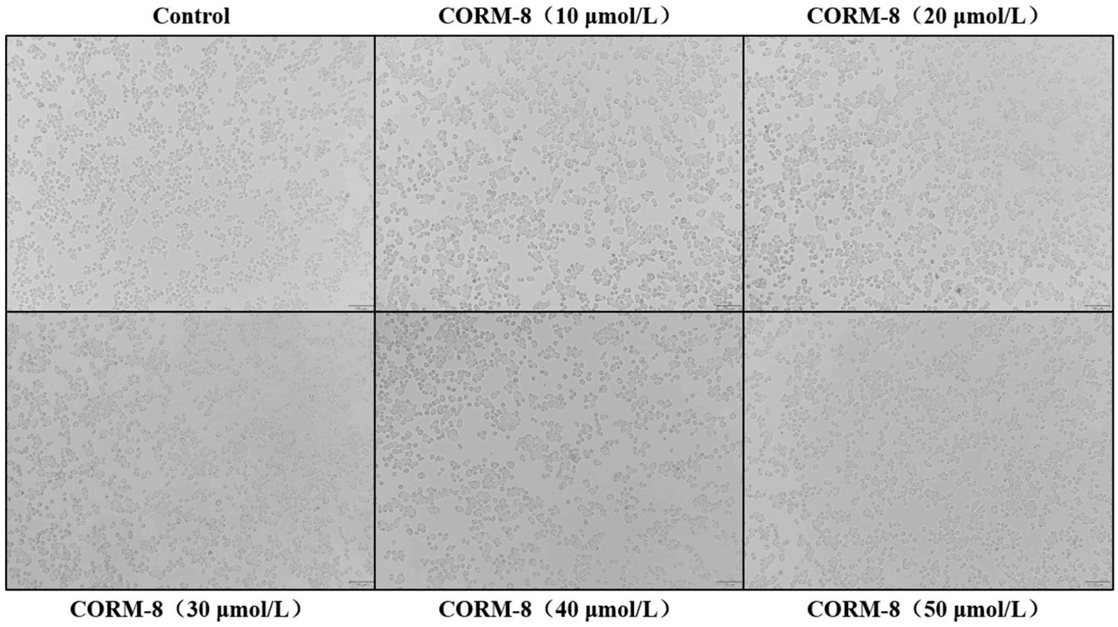

Figure S18 HT29 cell changes inhibited by **8**

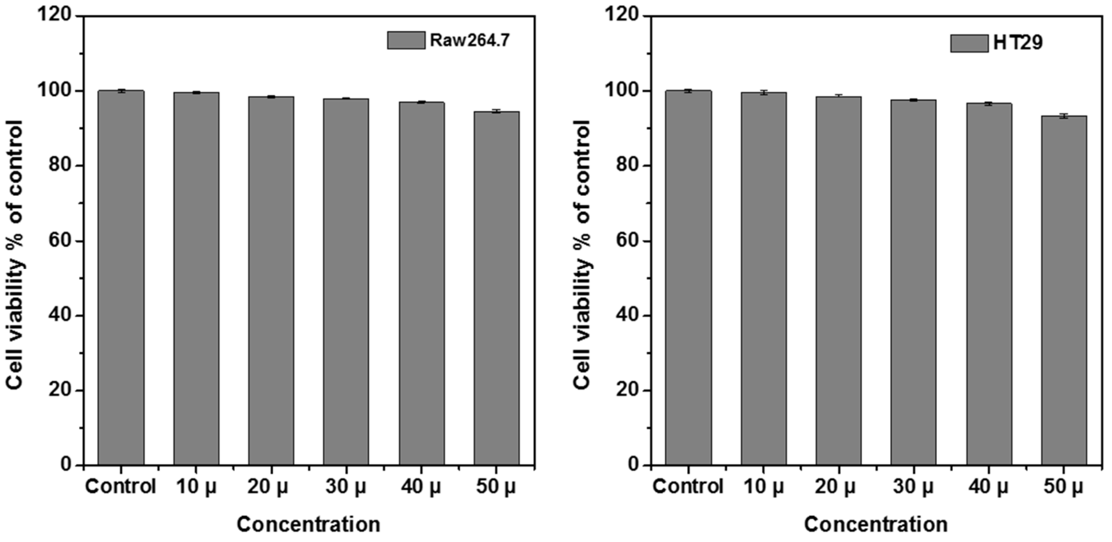

(a) (b)

Figure S19 Cell viability of RAW264.7 cell (a) and HT29 (b) in presence of **8**. Cells were grown in the presence of **8** (10-50 μM) and irradiated at 365 nm for 20 min.

Reference:

[1] SAINT; Data Reduction Software; Bruker AXS: Madison; WI; USA. 2009.

[2] G.M. Sheldrick, 2010, SADABS, Program for Empirical Absorption Correction of Area Detector Data. University of Gottingen, Gottingen, Germany.

[3] Dolomanov, O.V., Bourhis, L.J., Gildea, R.J, Howard, J.A.K. & Puschmann, H. (2009), J. Appl. Cryst. 42, 339-341.
